# Activity and structure of human (d)CTP deaminase CDADC1

**DOI:** 10.1073/pnas.2424245122

**Published:** 2025-05-05

**Authors:** Anton Slyvka, Ishan Rathore, Renbin Yang, Olga Gewartowska, Tapan Kanai, George T. Lountos, Krzysztof Skowronek, Mariusz Czarnocki-Cieciura, Alexander Wlodawer, Matthias Bochtler

**Affiliations:** ^a^Laboratory of Structural Biology, International Institute of Molecular and Cell Biology in Warsaw, Warsaw 02-109, Poland; ^b^Center for Structural Biology, National Cancer Institute, NIH, Frederick, MD 21702; ^c^Cancer Research Technology Program, Leidos Biomedical Research Inc., Frederick, MD 21701; ^d^Genome Engineering Facility, International Institute of Molecular and Cell Biology in Warsaw, Warsaw 02-109, Poland; ^e^Basic Science Program, Frederick National Laboratory for Cancer Research, Frederick, MD 21702; ^f^Biophysics Facility, International Institute of Molecular and Cell Biology in Warsaw, Warsaw 02-109, Poland; ^g^Laboratory of Protein Structure, International Institute of Molecular and Cell Biology in Warsaw, Warsaw 02-109, Poland; ^h^Institute of Biochemistry and Biophysics, Warsaw 02-106, Poland

**Keywords:** cytidine deaminase, nucleotide metabolism, cryoelectron microscopy, triphosphate

## Abstract

Vertebrates have evolved an understudied protein termed CDADC1 (NYD-SP15) that contains an inactive N-terminal and active C-terminal DCTD-like domain. Here, we show that human CDADC1 is a (d)CTP-specific deaminase, with a roughly 2-fold in vitro preference for dCTP over CTP. We determined high-resolution cryo-EM structures of CDADC1 in the absence of substrate and in complex with dCTP and 5-methyl-dCTP. The structures show that CDADC1 forms trimers and dimers of trimers in solution. The (d)CTP substrate is selected by a narrow pocket for the cytosine base and multiple lysine and arginine contacts to the triphosphate. Substrate binding promotes the association of trimers into hexamers and the transition of the hexamers from a loose to a tighter arrangement. Genetic experiments in mice show that loss of Cdadc1 is surprisingly well tolerated, even in the absence of the dCMP deaminase Dctd that is considered as the main source of dUMP, the precursor of dTTP.

## Significance

CDADC1 has evolved in vertebrates, presumably from a dCMP deaminase (DCTD)-like precursor. We show that CDADC1 has (d)CTP deaminase activity previously known only from prokaryotic and phage biology, where it is catalyzed by enzymes of a different architecture. The role of CDADC1 in vertebrate biology remains unclear. In standard laboratory conditions, Cdadc1 loss is surprisingly well tolerated in mice, even in the absence of the dCMP deaminase Dctd.

The balance between amino- and keto-pyrimidine 2′-deoxynucleotides and ribonucleotides is carefully regulated by anabolism, salvage, catabolism, and utilization (1). Deaminases catalyze the conversion from (2′-deoxy)cytidines to the corresponding (2′-deoxy)uridines. The phosphorylation state in which dC is deaminated and the enzymes that catalyze the reaction differ significantly across species. Gram-negative bacteria and some archaea utilize dCTP deaminases (DCDs) that produce dUTP, which is then dephosphorylated to dUMP by dUTPases (DUTs) (2). DCDs can be either monofunctional (e.g. EC 3.5.4.13) (3) or bifunctional DCD-DUTs (EC 3.5.4.30) (2–4). Both groups deaminate dCTP in a zinc-independent fashion, utilizing two water molecules for catalysis (5). The enzymes form homotrimers (DCD) or dimers of trimers (DCD-DUT) and their active sites are located at the interface of two protomers within a trimer.

In gram-positive bacteria and eukaryotes, dCMP is directly converted to dUMP by zinc-dependent deoxycytidylate deaminases (DCTDs, EC 3.5.4.12), which are not related to DCDs by sequence, structure, or catalytic mechanism. Every reported DCTD is a ring-shaped homohexamer (protomer size ~20 kDa). Each protomer harbors a single CMP/dCMP deaminase-like domain (IPR002125) that contains one catalytic site and one allosteric site. The catalytic site features the characteristic HxE and PCxxC motifs. Two cysteines, one histidine, and one water molecule tetrahedrally coordinate a zinc ion. The glutamate residue is essential for catalysis and serves as a proton shuttle (6). The allosteric site of DCTDs, featuring the conserved G(Y/W)N(A/G) motif, is responsible for the binding of modulators: dCTP (activator) and dTTP (inhibitor). The extent of the response to modulators differs between the enzymes. For comparison, in the absence of dCTP, phage T4 DCTD is completely inactive, and human DCTD has at least 10-fold lower activity (7, 8).

DCTD is also active on 5-methyl-dCMP (^5m^dCMP) and 5-hydroxymethyl-dCMP (^5hm^dCMP), but it is inactive on CMP and CDP, suggesting a strong preference for the 2′-deoxycytidine (8, 9). Logically, DCTD enzymes do not deaminate their activator dCTP. However, two exceptions to this rule have been reported, both found in viruses. First, DCTD from *Paramecium bursaria* Chlorella virus 1 (PBCV-1) was shown to deaminate both dCDP and dCTP, albeit with lower efficiency than dCMP (10). The enzyme also exhibits an allosteric response to both dCTP and dTTP and displays positive cooperativity (10, 11). Second, DCTD from *Xanthomonas oryzae* phage XP-12 specifically deaminates ^5m^dCTP to produce dTTP independently from the host (12, 13). Although little is known about XP-12 DCTD biochemistry, it is clear from its sequence that the enzyme contains all features of zinc-dependent deaminases (14).

Vertebrates possess a second DCTD-like protein, CDADC1 (NYD-SP15) (15). Unlike DCTD, CDADC1 contains two CMP/dCMP-like deaminase domains (IPR002125), connected by a long (>100 residues) linker within a single polypeptide chain, resulting in CDADC1 being approximately triple the length of DCTD (e.g. 514 residues in human CDADC1 versus 178 in human DCTD). Both domains contain PCxxC motifs, likely responsible for zinc coordination. The C-terminal deaminase domain (CTD) of CDADC1 contains a potentially active HAE motif, but its N-terminal domain (NTD) appears to be naturally inactivated due to the mutation of the proton shuttle glutamate (the HAG motif instead of HAE), suggesting noncatalytic function. The knowledge of CDADC1 is extremely limited. High levels of its mRNA were found in adult mouse and human testes, suggesting a possible role in testicular development and spermatogenesis (16). The follow-up study reported that CDADC1 can shuttle between cytoplasm and nucleus in a cell cycle-dependent manner. Based on the yeast two-hybrid assay, the authors suggested that CDADC1 forms a homodimer (15).

Here, we describe a comprehensive molecular, biochemical, and structural investigation of human CDADC1. We show that the enzyme deaminates (d)CTP and report cryo-EM structures of CDADC1 trimers and hexamers in the free form, as well as with dCTP or ^5m^dCTP substrates. The structures explain the CDADC1 preference for triphosphorylated (2′-deoxy)nucleosides. Moreover, we report genetic studies in mice that show that Cdadc1 loss is well tolerated, even in combination with the Dctd dCMP deaminase loss.

## Results

### Biochemical Characterization of CDADC1.

**Fig. 1.**
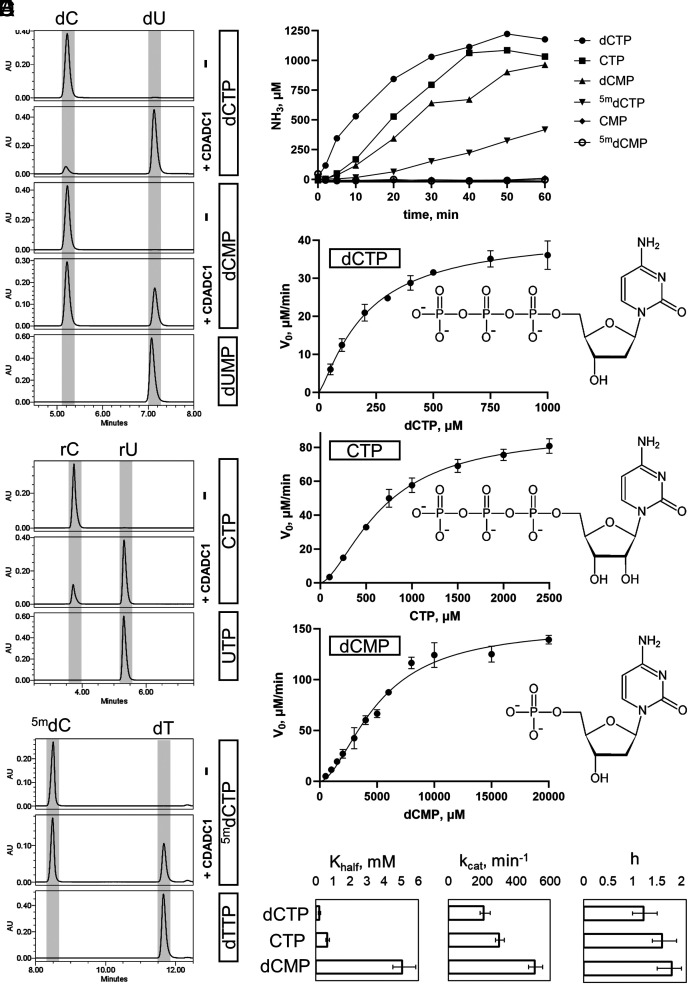
In vitro activity of human CDADC1. (*A*) UHPLC traces of substrates treated with CDADC1. (Deoxy)nucleotides were dephosphorylated prior to the injection on the column. Dephosphorylated dUMP, UTP, and dTTP were used as deamination references for dCTP/dCMP, CTP, and ^5m^dCTP, respectively. (*B*) Kinetic measurement of the CDADC1 deaminase activity on different substrates using fluorescent detection of ammonia. CDADC1 was used at 0.5 μM (monomer), and each substrate was used at 1 mM in High Salt Reaction Buffer. (*C*–*E*) Initial deamination velocities (V_0_) plotted against the concentration of dCTP, CTP, and dCMP, respectively. The curves represent the fit into the allosteric sigmoidal model. V_0_ values were measured in at least three independent reactions and the error bars represent SD. CDADC1 concentration was 0.2 μM (dCTP) or 0.3 μM (dCMP and CTP) in Low Salt Reaction Buffer. Chemical drawing of each substrate is provided on the right side of the corresponding graph. (*F*) Bar plot comparison of the kinetic parameters (K_half_, k_cat_, and h) of CDADC1 for each substrate. Error bars represent the 95% CI.

In the absence of prior knowledge of CDADC1 biochemistry, we started our experimental work from the ground up. We cloned and overexpressed human CDADC1 in *Escherichia coli* and developed a purification protocol that allowed us to obtain a highly homogeneous protein (*SI Appendix*, Fig. S1). Next, we used purified CDADC1 to conduct an ultrahigh performance liquid chromatography (UHPLC)-based screen for its potential substrate(s) (Fig. 1*A*, and *SI Appendix*, S2). The test set included nucleobases, (deoxy)nucleosides, and mono- and triphosphorylated (deoxy)nucleotides with primary amino group that could in principle be deaminated. After incubation with CDADC1, the reaction products were treated with the degradation-dephosphorylation mix [prepared according to (17)] and resolved on a reverse phase UHPLC column. As expected for a DCTD-related enzyme, CDADC1 could deaminate dCMP. However, to our surprise, dCTP and CTP were much better substrates (Fig. 1*A*). CDADC1 had a significantly reduced activity on ^5m^dCTP and only trace activity on ^5hm^dCTP and CMP (Fig. 1*A* and *SI Appendix*, Fig. S2*A*). The enzyme was inactive on other tested compounds, including ^5m^dCMP (*SI Appendix*, Fig. S2*B*). The same activity pattern (dCTP > CTP > dCMP > ^5m^dCTP >> ^5hm^dCTP > CMP) was observed in the *o*-phthalaldehyde-based ammonia detection assay (Fig. 1*B*). The results clearly suggested that CDADC1 had a preference for the triphosphorylated (deoxy)cytidines. Also, the more severe drop of activity between CTP and CMP than between dCTP and dCMP suggested that CDADC1 preferred 2′-deoxycytidine nucleotides. Low activity on ^5m^dCTP and only trace activity on ^5hm^dCTP suggested that C5 modification was sterically unfavorable. To test whether the HAE motif in the CDADC1 CTD was solely responsible for the activity we mutated the E400 residue to alanine (E400A). The purified CDADC1 E400A variant was inactive in vitro (*SI Appendix*, Fig. S3), confirming that the NTD was not catalytically active and likely had a noncatalytic role.

Deamination of dCMP by every DCTD is activated by dCTP and inhibited by dTTP in the presence of Mg^2+^ ions (18). Amino acid sequence comparison of CDADC1 with diverse DCTDs indicated the presence of the putative allosteric site in the active domain CTD (G365-YN-A368), but not in the NTD (*SI Appendix*, Fig. S4). Despite the presence of this putative allosteric site, neither dCTP nor dTTP affected the (weak) dCMP deaminase activity of CDADC1, regardless of Mg^2+^ presence (*SI Appendix*, Fig. S5 *A* and *B*). Moreover, CDADC1 activity on dCTP was also not affected by dTTP, UTP, dTMP, or CMP (*SI Appendix*, Fig. S5*C*). Since CDADC1 behaved more like a DCD or DCD-DUT than like a DCTD enzyme with respect to allosteric regulation, we wondered whether CDADC1 may have phosphatase activity in addition to deaminase activity, like DCD-DUT. However, this possibility was ruled out, because CDADC1 treated dCTP (with UV properties like uridine) bound anion exchange resin as strongly as untreated dCTP (*SI Appendix*, Fig. S6).

To understand CDADC1 substrate preferences, we measured steady-state enzymatic kinetics with dCTP, CTP, and dCMP. The original plan also included ^5m^dCTP, but the commercially available amounts were impractical for achieving CDADC1 saturation (*SI Appendix*, Fig. S7). We employed fluorescent detection because it allowed for an accurate quantification of the absolute amounts of ammonia released in deamination. First, we established the dynamic range of CDADC1 concentration for every tested substrate (*SI Appendix*, Fig. S8). We then measured the initial CDADC1 velocities (V_0_) of each substrate at a minimum of eight concentrations (Fig. 1*B*). Plotting V_0_ against the corresponding substrate concentration did not produce the typical hyperbolic Michaelis–Menten (M-M) curves. With CTP and dCMP the curves were clearly S-shaped, and slightly with dCTP, which suggested a substrate-dependent cooperativity (Fig. 1 *C*–*E*). Indeed, data analysis in both M-M and cooperative models showed that CTP and dCMP data were better fitted by the latter, with Hill coefficients (*h*) of 1.6 and 1.8, respectively. For dCTP, the fitted *h* was only 1.22, indicating weak or no cooperativity for this substrate (*SI Appendix*, Fig. S9). Having established the model, we were able to determine the K_half_ values, the M-M constant (K_M_) equivalents in the cooperative model, for each substrate. The K_half_ for dCTP is the lowest (0.2 mM), for CTP it is more than threefold higher (0.7 mM), for dCMP it is very high (~5 mM), explaining the CDADC1 preference for dCTP. Additionally, CDADC1 achieves its maximum velocity (V_max_) at a much lower concentration of dCTP than CTP and dCMP. However, the apparent catalytic constant (k_cat_) for dCTP (3.5 s^−1^) is actually lower than for CTP (5.0 s^−1^) or dCMP (8.5 s^−1^) (Fig. 1*F*), which was tentatively attributed to slower product release for the tighter-binding substrate.

We conclude that CDADC1 is most active on dCTP and that any modifications (2′-OH, C5 substituents, fewer phosphates) lower its activity by reducing substrate binding. Although CDADC1 shares the dCTP deaminase activity with prokaryotic DCDs and DCD-DUTs, it differs with respect to domain architecture and regulation.

### The Overall Structure of Human CDADC1.

**Fig. 2.**
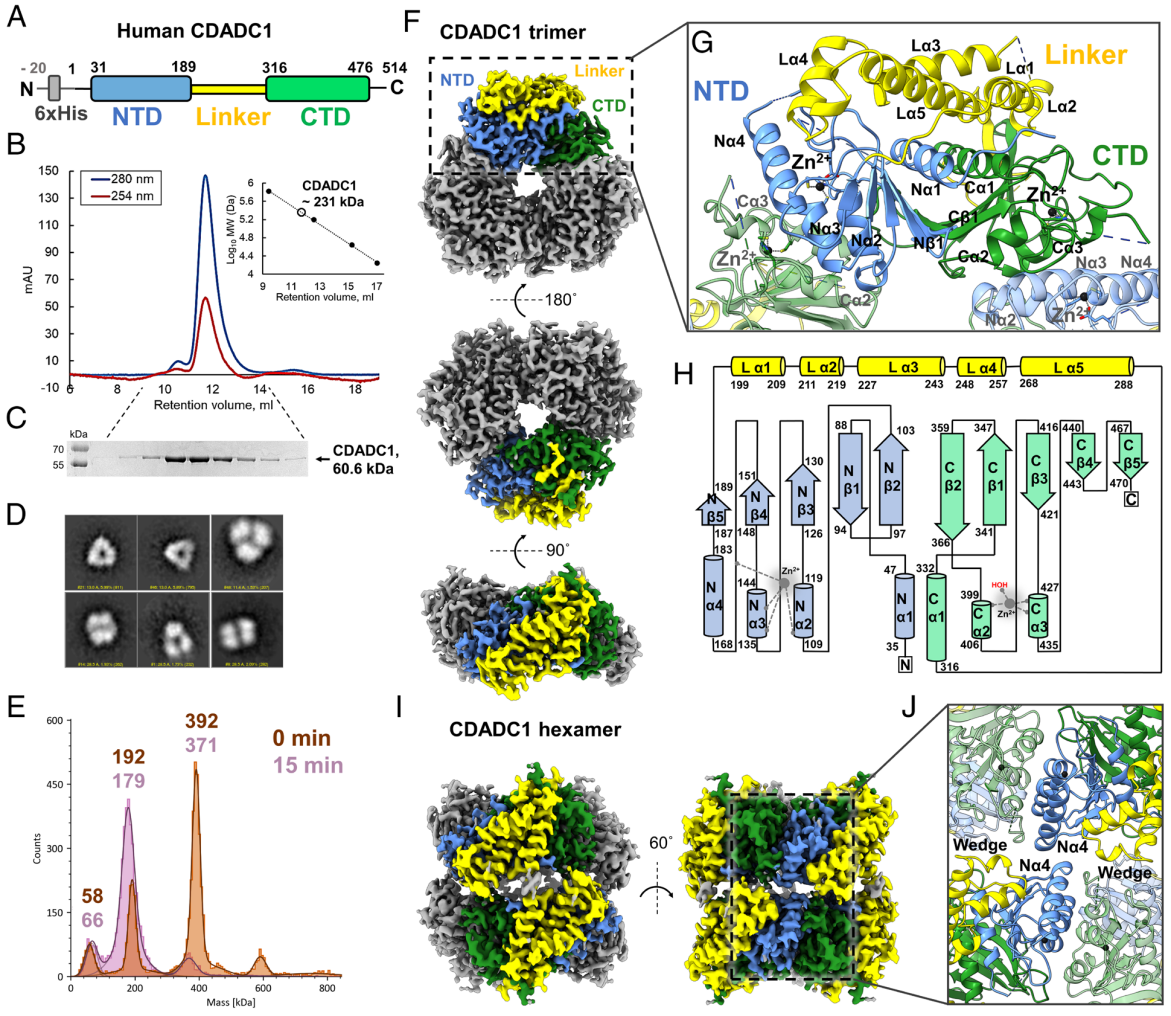
Overall structure of human CDADC1. (*A*) Schematic of the domain organization of CDADC1. NTD (blue) is the N-terminal catalytically inactive deaminase domain, and CTD (green) is the C-terminal catalytically active deaminase domain. (*B*) Analytical size-exclusion chromatography profile of purified CDADC1. The inset shows CDADC1 molecular weight estimation (empty circle) using calibration standards (black dots). Standards used (*Left* to *Right*): thyroglobulin (670 kDa), γ-globulin (158 kDa), ovalbumin (44 kDa), and myoglobin (17 kDa). (*C*) SDS-PAGE analysis of the analytical size-exclusion fractions. (*D*) Selected 2D classes of the ns-EM images of CDADC1. (*E*) Estimation of CDADC1 oligomeric states using mass-photometry. (*F*) ESP map of CDADC1 trimer in three orientations. The map of one protomer is colored according to panel *A*. (*G*) Close-up view on the structure of CDADC1 protomer and its contacts with neighboring protomers. (*H*) 2D diagram of CDADC1 protomer. For both panels *G* and *H*, α and β stand for the α-helix and β-strand, respectively. Letters N, L, and C stand for the NTD, linker, and CTD respectively. (*I*) ESP map of CDADC1 hexamer in the absence of substrate in two orientations. Two protomers from dimerizing trimers are colored as above. (*J*) Close-up view on the contacts within the hexamer.

The unusual biochemical properties of CDADC1 prompted us to investigate its structure. The predicted molecular mass of the single polypeptide of human CDADC1 is ~58.5 kDa (~60.6 kDa with N-terminal 6xHis tag) (Fig. 2*A*) and, as predicted, it migrates accordingly on SDS-PAGE (Fig. 2*C*). However, purified CDADC1 migrated on a size exclusion chromatography (SEC) with a retention time characteristic for a ~230 kDa protein (Fig. 2*B*). The retention time was consistent with a tetramer or a mixture of other oligomeric forms. Negative stain electron microscopy (ns-EM) indicated that CDADC1 molecule exhibits three-fold symmetry, with a particle diameter of 10 to 13 nm, suggesting that it is a trimer, hexamer, or a mix of both (Fig. 2*D*). Further analysis using mass photometry (MP) supported the ns-EM result, by showing that CDADC1 indeed exists in solution as both trimer (~190 kDa) and hexamer (~390 kDa) (Fig. 2*E*). Importantly, we observed that the hexamers dissociated to trimers and even to monomers over time, presumably due to the low protein concentration used for MP (~0.06 μM, monomer). We concluded that CDADC1 hexamers are in fact dimers of trimers and that the ~230 kDa SEC peak represents the dynamic equilibrium between the two oligomeric states.

Encouraged by these observations, we subjected CDADC1 to single-particle cryoelectron microscopy (cryo-EM). We collected datasets of the CDADC1 E400A variant alone, in the presence of dCTP (“good” substrate), and in the presence of ^5m^dCTP (“bad” substrate) (*SI Appendix*, Table S1). All three datasets were analyzed imposing either C3 (trimer) or D3 (dimer of trimers, hexamer) symmetry (*SI Appendix*, Table S1). Extensive processing enabled us to reconstruct high-resolution electrostatic potential (ESP) maps (2.4 to 3.0 Å) (*SI Appendix*, Figs. S10 and S11 and Table S1). The trimer maps in the ligand binding region and the trimer conformation suggested a ligand-free state of the trimer, irrespective of whether or not dCTP or ^5m^dCTP was present in the sample (*SI Appendix,* Figs. S12 and S13). Therefore, only four models were submitted to PDB, a trimer without ligand (PDB: 9HFQ) (Fig. 2 *F*–*H*), a hexamer without ligand (PDB: 9HFR) (Fig. 2 *I* and *J*), a hexamer with bound dCTP (PDB: 9HFS), and a hexamer with bound ^5m^dCTP (PDB: 9HFT). The hexamer models were similar but differed in the interface region. Trimers were more tightly packed against each other in the dCTP and ^5m^dCTP bound states than in the state without substrate (*SI Appendix,* Fig. S14). Together, the data are consistent with a model that ligand binding promotes the formation of hexamers, at least in the protein concentration range used in the cryo-EM experiments.

### Cryo-EM Structures of CDADC1 in the Absence of a Ligand.

#### Protomer.

As expected, each CDADC1 protomer consists of two deaminase domains, N-terminal (NTD) and C-terminal (CTD) (Fig. 2*G*). Our structures show that the NTD and CTD span the residues 31–189 and 316–476, respectively, slightly different than automatically annotated (ProRule PRU01083). Two domains are positioned in an antiparallel back-to-back configuration relative to each other. They are connected by a long linker (residues 190–315), composed of five α-helices, one of which (L (linker) α5) bears a putative nuclear export signal (residues 271–283). Importantly, the peptide bond between N266 and P267 upstream of the Lα5 has a cis-conformation, resulting in a wedge-like structure of the region (residues 262–272). In addition to the linker, the two domains are connected by reciprocal interactions between their α1 helices and β2 strands. Both NTD and CTD contain a standard cytidine deaminase core (19). It consists of a five-stranded mixed β-sheet (β2-β5 parallel, β1 antiparallel), sandwiched between three α-helices, α1 from one side and α2 and α3 from the other (Fig. 2*H*). The NTD, but not CTD, contains an additional α-helix (N (NTD) α4, residues 168–183), which is also present in human DCTD (PDB 2W4L). Consistent with the intrinsic disorder prediction (*SI Appendix,* Fig. S15), every CDADC1 protomer contains unstructured regions, which are undefined in the ESP maps. These regions include residues 1–28 upstream of the NTD, 53–83 and 163–166 within the NTD, 221–224 within the linker, 304–309 and 376–390 within the CTD, and 477–514 downstream of CTD. The latter region contains the putative bipartite nuclear localization signal (488–510).

**Fig. 3.**
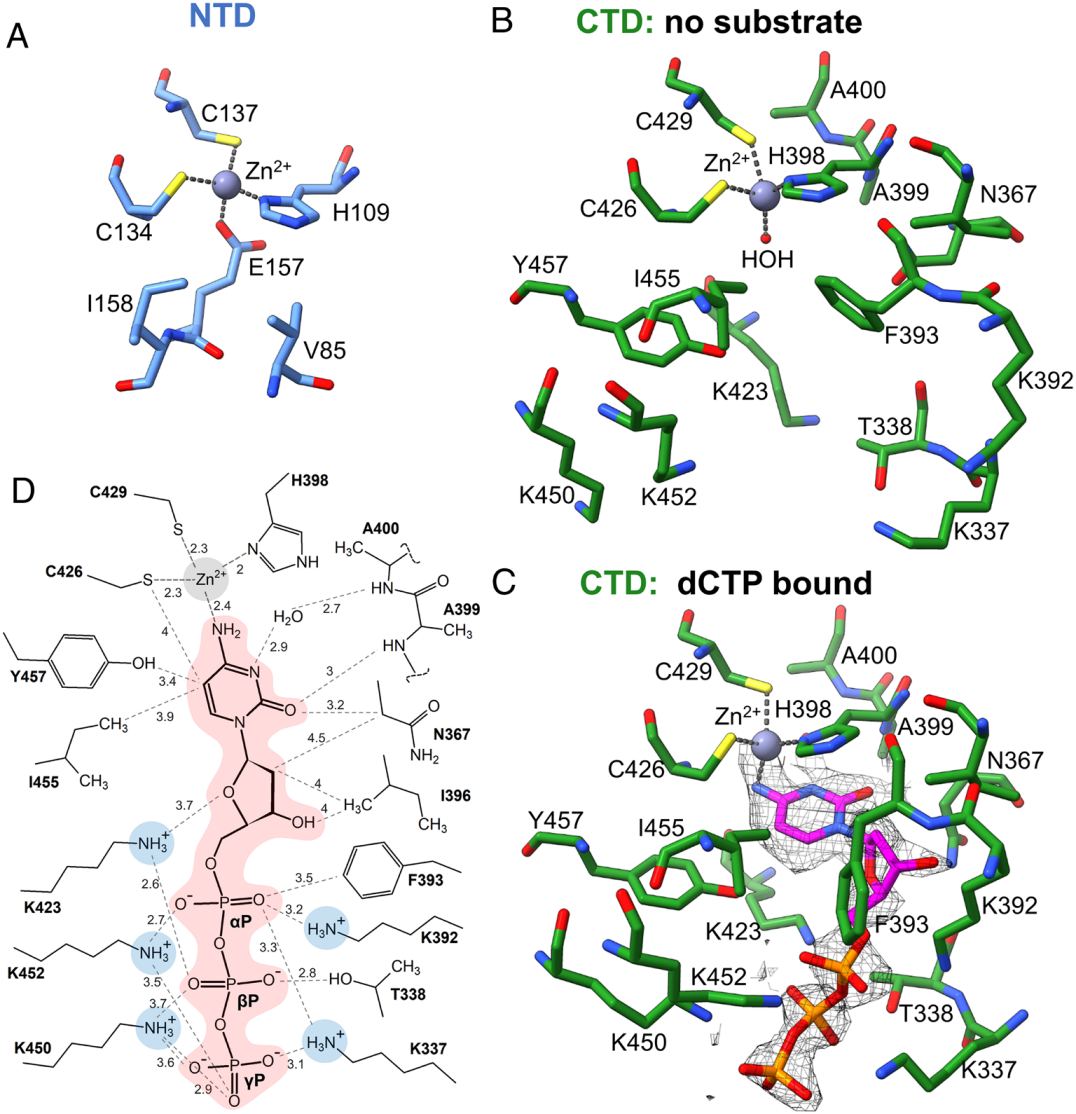
Substrate binding by CDADC1. (*A*) The inactive site in the CDADC1 NTD. (*B*) Active site of the CDADC1 CTD in the absence of substrate. (*C*) Active site of the CDADC1 CTD with bound dCTP. The ESP map for dCTP is shown as mesh. (*D*) 2D diagram of the interactions between dCTP and CDADC1 CTD active site residues. For simplicity, all noncovalent interactions are shown as dashed lines. The distances are in Å.

NTD and CTD tetrahedrally coordinate one zinc ion (Zn^2+^) each using conserved PCxxC motifs (Figs. 2*G* and 3 *A* and *B*). In the NTD, Zn^2+^ is coordinated by H109, C134, C137, and surprisingly not by a water molecule, but by a side chain carboxyl of E157, which blocks the access to the Zn^2+^. The remaining pocket space is fully occupied by V85 and I158. The inactive HAG sequence is located in the same spot in the NTD as the conserved HAE motif in every DCTD, strengthening the hypothesis that G111 is a result of natural mutation during evolution. Therefore, our structures demonstrate that NTD is inactivated in two different ways (Fig. 3*A*). In the CTD, Zn^2+^ is coordinated by C246, C429, H398, and one water molecule, which together with I455 and F393 form the ceiling of the active site. The bottom of the active site is formed by hydrophobic residues V421, V340, G341, and A342. The left wall is defined by the main chains of C424 and P425 and a side chain of Y457. The right wall is made by the main chains of H398 and A399 of the conserved HAE motif (20) (Fig. 3*B*). E400 is an essential residue (*SI Appendix,* Fig. S3), that in many deaminases acts as a proton shuttle (Fig. 3*B*) (6). In our structures, the side chain of E400 is missing, because the residue was mutated to alanine to prevent catalysis.

In addition to the catalytic site, DCTDs have an allosteric site that regulates their activity. Since DCTD proteins have only one domain, their allosteric site is located at the interface between the protomers in the hexamer. The binding of dCTP was shown to stabilize hexamers and is believed to be the main mechanism of allosteric regulation (21, 22). A potentially equivalent site to the allosteric site in the DCTDs is present in the CTD, but not in the NTD of CDADC1 (*SI Appendix,* Fig. S16). In agreement with our inability to identify an allosteric regulator biochemically, the CDADC1 maps suggest that this putative allosteric site is not occupied. Thus, a fusion of NTD and CTD in CDADC1 may have permanently stabilized the interface between the two and made the canonical allosteric regulation obsolete.

#### Trimer.

CDADC1 trimers are closed rings formed by three protomers assembled in a head-to-tail orientation. The majority of the contacts are formed between Nα2 and Nα3 from the NTD of one protomer and Cα2 and Cα3 of the CTD of the other. In addition, the structured portion of the loop 367–396 (369–375 and 391–396) near the CTD active site forms contacts with both Nα3 and Nα4 from the NTD. This results in the vast interface of ~1,100 Å^2^ between the two adjacent protomers, according to the PISA server (23). This also means that the NTD remains an integral structural component of CDADC1 even though it has lost its catalytic function.

#### Hexamer.

CDADC1 hexamers are formed by symmetric stacking of two trimers, i.e. trimers face each other with the same sides. Each protomer equally contributes to the hexamer formation. CDADC1 apo hexamers are less tight than trimers, with a contact area per protomer of ~270 Å^2^. Each protomer from one trimer interacts with two protomers from the other trimer. With one of them, it is arranged in an antiparallel symmetric manner. However, no direct contacts can be seen between the two protomers in the apo model. One potential contact point between the pair is the “blob” in the map where two trimers are the furthest from each other (~7 Å), between two C197. The blob is likely derived from the partially structured 1–28 region (*SI Appendix,* Fig. S17). We exclude the possibility of potential disulfide bond formation with other residues because none of the missing regions of this intracellular protein contains cysteines. The interaction with the second protomer is more direct. It is achieved by symmetric hydrogen bonds between guanidino groups of R185 and carbonyl oxygens of A186, located between Nα4 and Nβ5 of the NTD. Therefore, we conclude that this interaction is likely the dominant force for hexamer formation in the absence of substrate, which highlights the important role played by Nα4 in both trimer and hexamer formation.

### Cryo-EM Structures of CDADC1 with dCTP and ^5m^dCTP.

#### Active site with dCTP.

We also calculated ESP maps and built models of trimeric and hexameric CDADC1 particles in the presence of dCTP and ^5m^dCTP. CDADC1 trimers that were observed in samples containing dCTP or ^5m^dCTP do not differ much from the apo trimers. There is no evidence for ligand binding in the maps of the CTD active sites, and no significant structural changes seem to occur (*SI Appendix,* Figs. S12 and S13), suggesting that trimers do not bind dCTP or ^5m^dCTP. However, CDADC1 hexamers reconstructed from the same datasets are different. In the presence of dCTP, each CDADC1 protomer contains a well-defined dCTP in the active site, therefore one CDADC1 hexamer binds six dCTP molecules. Consistent with our biochemical results, the maps for the allosteric site indicate that neither dCTP nor ^5m^dCTP are bound, suggesting that the observed cooperativity must originate from the active site (*SI Appendix,* Fig. S16). Within the active site, the cytosine base of dCTP stacks under Zn^2+^ coordinating H398. Its O2 enforces the rotation of the sidechain of N367 and forms a hydrogen bond with NH of A399. The rotated sidechain of N367 allows for accommodation of both 2′C and 3′OH. This positions the N3–N4 edge of the base toward the usual location of the catalytic glutamate (E400 in CDADC1), a configuration shared among all Zn^2+^-dependent cytidine deaminases (6). Since we replaced E400 with alanine, the space normally filled by the sidechain is occupied by a water molecule. In the absence of E400, dCTP N4 displaces the Zn^2+^ bound water molecule of the ligand-free structures and participates in the Zn^2+^ coordination, mimicking the transition state. The C5 of dCTP is located at <4 Å from C426, I455, and Y457, strongly suggesting that the low CDADC1 activity on ^5m^dCTP and ^5hm^dCTP is a result of a steric conflict. Accommodation of dCTP also drives the significant rearrangement of the K392-F393 region, likely due to the steric conflict between the 3′OH and α-phosphate (αP) on the dCTP side and aromatic ring of the F393 on the CDADC1 side. F393 remains in close proximity to the αP (3.5 Å), and thus it seems to act as a “sensor” of the bound substrate. The 2′C of the bound dCTP is brought into close proximity (~4 Å) of the side chain of I396, suggesting that the presence of a hydroxyl group would be sterically unfavorable. This explains the lower CDADC1 activity on CTP than on dCTP.

The lack of activity on CMP but not on dCMP suggests that the interaction with all three phosphates is necessary to force the ribose into the active site. Indeed, the CDADC1 structure with bound dCTP demonstrates an extensive network of interactions with the triphosphate tail. Astonishingly, five lysine residues that constitute the positively charged patch on the CDADC1 surface all rearrange to form salt bridges with dCTP phosphates. The K392–F393 rearrangement mentioned earlier enables the Nε of K392 to form a salt bridge with the αP. K423 forms a salt bridge with βP, and possibly also a hydrogen bond with the 4’O of the deoxyribose. K337 and K452 form salt bridges with both αP and γP from two opposite sides. K450 forms an additional salt bridge with γP (Fig. 3 *C* and *D*). All five lysine residues that interact with the dCTP or ^5m^dCTP triphosphate tail are highly conserved in CDADC1 proteins, from sharks all the way to humans (*SI Appendix,* Fig. S18). The multiple ionic interactions with dCTP contribute not only to ligand specificity, but also to long-distance communication within the CDADC1 hexamer. We have compared the positively charged ligand binding site of CDADC1 with the equivalent regions in XP-12 deaminase (a viral deaminase active on the triphosphate), PBCV-1 deaminase (another viral deaminase active on the monophosphate and triphosphate), and human DCTD (active on the monophosphate). As expected, the triphosphate-specific CDADC1 and XP-12 deaminase show the greatest accumulation of lysine and arginine residues (*SI Appendix,* Fig. S19).

#### Active site with ^5m^dCTP.

As could be expected based on the kinetic data (Fig. 1*F*), the ESP map for ^5m^dCTP is less clear than the map for dCTP. A comparison of the CDADC1 active sites with dCTP and ^5m^dCTP shows that the two substrates are bound similarly (*SI Appendix,* Fig. S20). However, the C5 methyl of the ^5m^dCTP clashes with the hydroxyl of Y457. To resolve the conflict, both Y457 and ^5m^dCTP shift away from each other, which puts N4 of ^5m^dCTP in the less favorable orientation for catalysis. Moreover, the shift results in the weakening of the electrostatic interactions between the triphosphate tail and the lysine residues. In particular, the salt bridge between the K423 and the αP seems to be disrupted almost completely. Since DCTDs usually do not select against C5-modified substrates, we compared the active sites of known DCTDs with the active site of CDADC1. As a control, we used the AlphaFold2 (24) model of phage XP-12 DCTD that specifically targets ^5m^dCTP. Indeed, DCTDs either have more space in the region (human DCTD) or feature an aromatic residue able to participate in the electron–π interaction with the methyl group (W121 in PBCV-1, W128 in XP-12, and Y153 in T4) (*SI Appendix,* Fig. S21). Therefore, the active site of CDADC1 has evolved to specifically bind the unmodified dCTP, possibly to avoid dTTP binding.

#### Substrate-induced rearrangement.

**Fig. 4.**
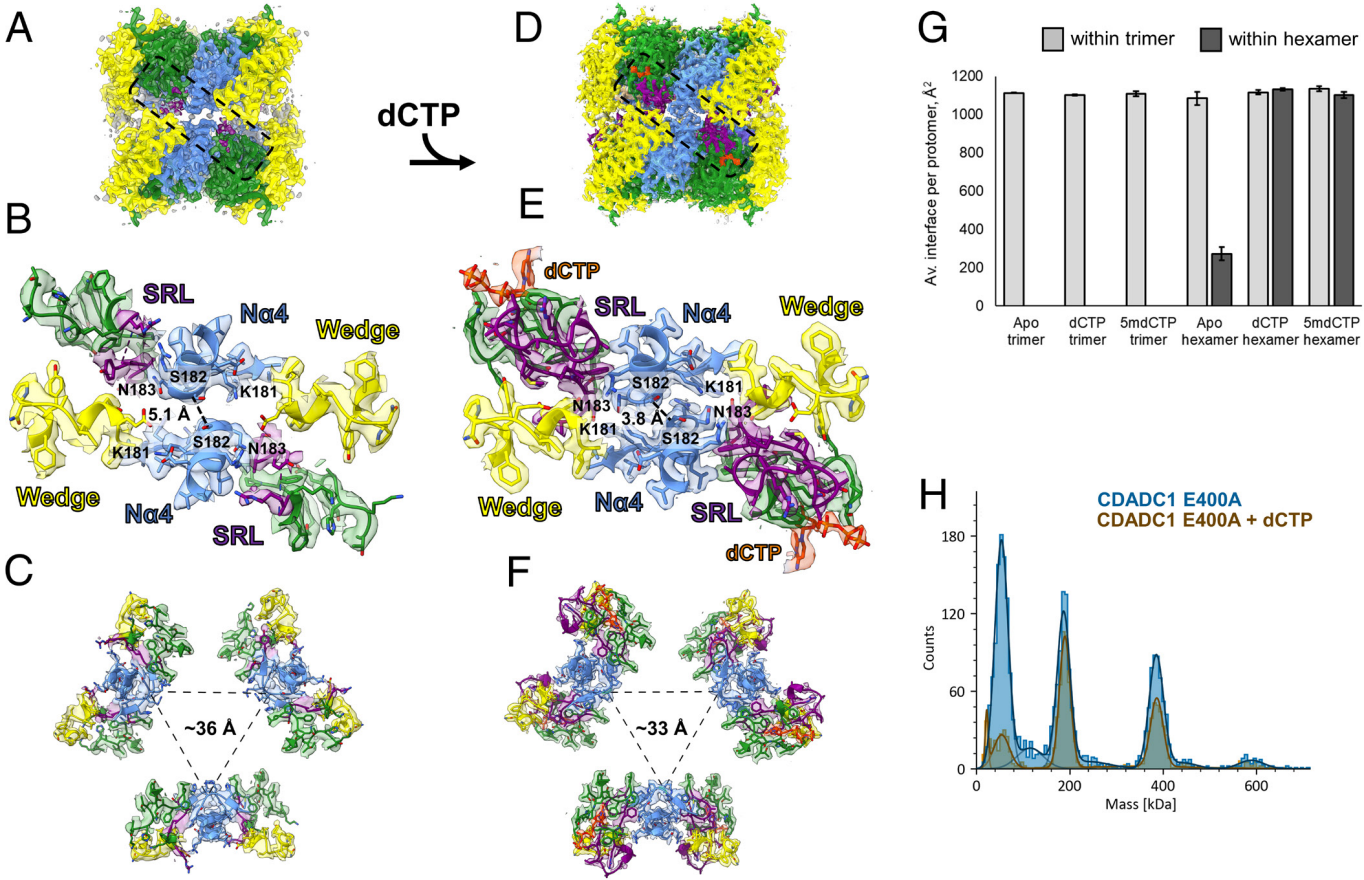
Substrate-induced structural rearrangements in CDADC1. (*A*–*C*) CDADC1 hexamer in the absence of the ligand. (*D*–*F*) CDADC1 hexamer with bound dCTP. (*A* and *D*) Side view of the cryo-EM maps of hexamers. ESP maps of the NTD, linker, and CTD in every protomer are colored blue, yellow, and green, respectively. Densities that correspond to dCTP and SRL are colored orange and purple, respectively. Dashed boxes highlight the interface region. (*B* and *E*) Focused view on the interface region without a ligand (*B*) and with bound dCTP (*E*). Models and corresponding ESP maps are colored as in *A* and *D*. Each interface is composed of contacts between four protomers that belong to two trimers. Distinct structural features are labeled in corresponding colors. For better visualization of the rearrangement, several amino acid residues are highlighted (K181, S182, and N183). Dashed lines represent distances (Å) between Cα’s of S182 of the opposing protomers. (*C* and *F*) Top view of the trimer–trimer interfaces, along the three-fold axis of symmetry. Dashed lines are drawn between Cα atoms of S182 that face the inner side of the hexamer. The mean distance is shown in Å. (*G*) Average interface area (Å^2^) per protomer in different CDADC1 models. Area was calculated using DALI server. Error bars represent SD. (*H*) Mass-photometry measurement of the effect of the ligand on CDADC1 oligomeric state. CDADC1 at 0.063 μM (monomer) without a ligand or in the presence of 1 mM dCTP at room temperature for 10 min.

The incorporation of dCTP into the active site triggers a cascade of structural changes on an increasing scale. The rotated N367 forms a hydrogen bond with T333, and T338 forms an additional hydrogen bond with dCTP βP. The loop region between these two threonines undergoes reorganization to avoid the collision between K393 and K377 bound to dCTP and H336. All the above leads to the stabilization of a large unstructured region (residues 376–390). This region is visible only in the EP maps of the CDADC1 hexamers with bound dCTP. A significant portion of this region (residues 381–390) folds around the sidechain of R391, in a C-shaped manner. The chain of electrostatic interactions: dCTP (αP and γP)–K452–D383–R391–D454–R288 also seems to contribute to the stabilization. Moreover, the upstream 373–375 region which is visible in the maps in the absence of substrate also rearranges. Therefore, for simplicity, the entire 373–390 region will be regarded as a substrate response loop (SRL) (Fig. 4). The dCTP-induced reorganization of SRL substantially increases the number of contacts between CDADC1 protomers within both trimers and hexamers. Within trimers, new contacts are made with the Nα4 of the adjacent protomer, which slightly increases the interface area. A much larger difference is observed in the interaction between protomers that belong to different trimers in the hexamer. Most importantly, the two antiparallel protomers that are too far from each other to form contacts in the absence of substrate, directly interact in the presence of dCTP. The structured SRL in CTD of each protomer makes an extensive set of contacts with the cis-P267 wedge of the opposing protomer. This promotes the interaction between the two NTDs, which form many symmetric contacts, such as the K181–N183 hydrogen bond (Fig. 4 *B* and *E*). The interaction between opposing protomers is strong enough to bring two trimers closer by ~1.3 to 1.8 Å (Fig. 4 and *SI Appendix*, S14). Moreover, the entire Nα4 helices shift toward the center of the hexamer (Fig. 4 *C* and *F*). Upon dCTP binding, the combined interface area in CDADC1 hexamer increases to ~1,130 Å^2^ per protomer. Thus, a single protomer with bound dCTP contributes several-fold more of its surface to the hexamer interface than without a ligand (Fig. 4*G*), explaining the stabilizing effect of dCTP against CDADC1 dissociation that was observed at the very low protein concentrations used for MP (Fig. 4*H*). CDADC1 hexamers with ^5m^dCTP are (almost) as compact as those with dCTP (Fig. 4 and *SI Appendix*, S14). Moreover, the interface area per promoter in the hexamer is also similar (Fig. 4*G*). However, the map quality for the ligand and SRL is lower, reflecting the weaker binding of ^5m^dCTP compared to dCTP.

In order to understand how CDADC1 structural rearrangements are related to the cooperativity, we compared CDADC1 to its closest relatives, the DCTD proteins. The architecture of the CDADC1 trimer, which consists of six deaminase domains (NCNCNC), is strikingly similar to the architecture of the DCTD homohexamers (*SI Appendix*, Fig. S22*A*). Interestingly, the DCTD protomers contain loops similar to the SRL (*SI Appendix*, Fig. S22*B*). These loops form symmetric contacts with the α4 helix of the neighboring protomers in the hexamer ring. The binding of dCTP in the allosteric sites is believed to stabilize this interaction and thus promote DCTD activity (25). Despite the striking similarity between DCTD hexamers and CDADC1 trimers, symmetric contacts are impossible in the latter, because NTD and CTD are not equivalent, and only one pair of contacts between Nα4 and CTD SRL is formed within the CDADC1 trimer. However, upon dimerization of CDADC1 trimers, two pairs of symmetric contacts between SRLs and the wedges are formed, restoring the communication between the active sites. This explains why CDADC1 hexamer, but not trimer is the active form of the enzyme and suggests a structural basis for the cooperativity.

#### No consistent upregulation of CDADC1 upon virus infection.

**Fig. 5.**
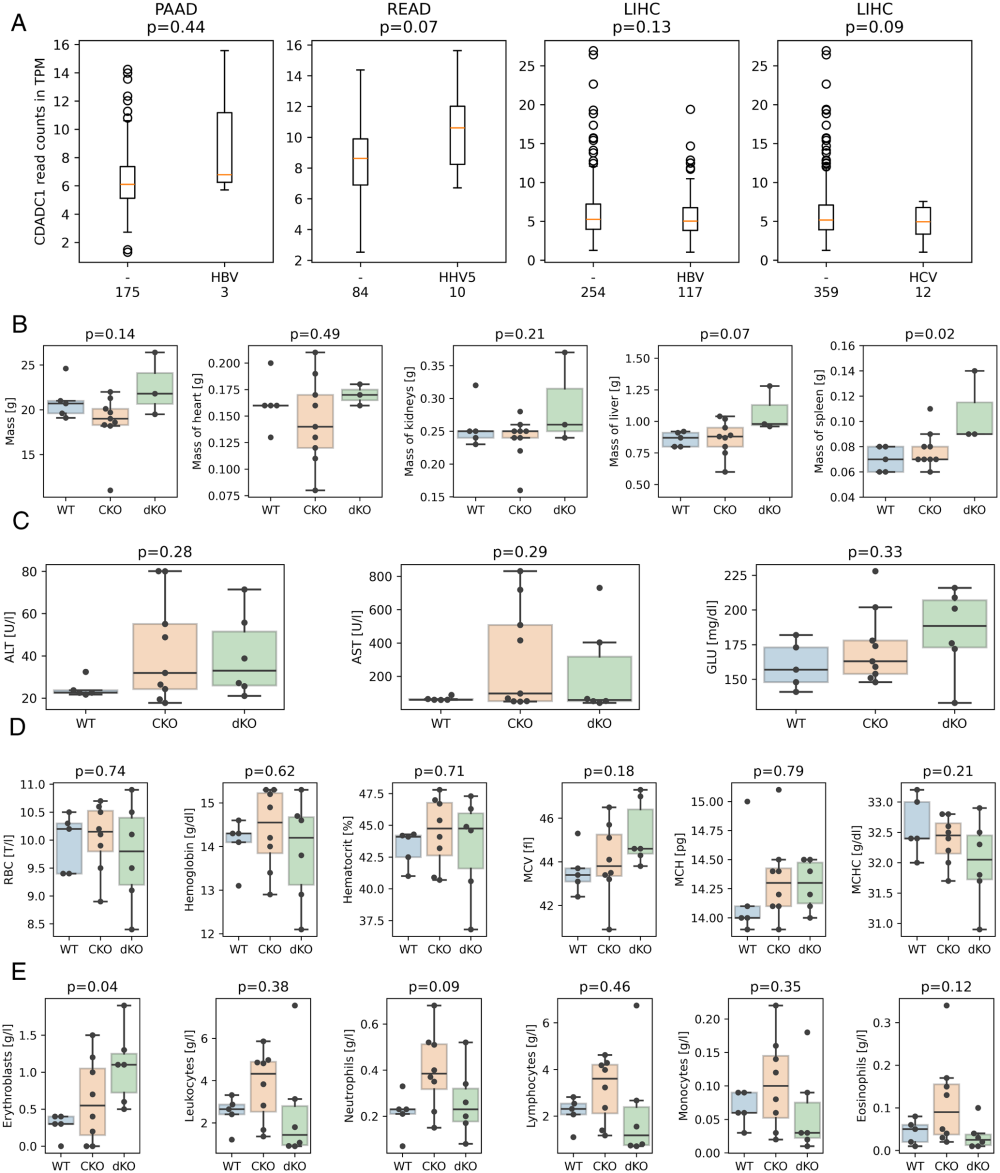
Lack of CDADC1 overexpression in response to viral infection in human cancer cell lines and effect of *Cdadc1* KO (CKO) and *Cdadc1/Dctd* double KO (dKO) in mice. (*A*) CDADC1 transcript levels from OncoDB, in transcripts per million (TPM), in virus-infected and noninfected human adenocarcinomas (PAAD: pancreatic adenocarcinoma, READ: rectum adenocarcinoma), and other malignancies (LIHC: liver hepatocellular carcinoma). HBV: hepatitis B virus, HCV: hepatitis C virus, HHV5: cytomegalovirus. Numbers below the panels indicate the number of samples, and p-values are for a two-sided *t* test (for unequal variances). (*B*) Effect of CKO and dKO on mouse whole body mass and organ weights (comparison only among females). (*C*) Effect of CKO and dKO on metabolic indicators (ALT: alanine aminotransferase, AST: aspartate aminotransferase, GLU: glucose). (*D*) Effect on red blood cells (comparison only among females, RBC: red blood cell count, MCV: mean corpuscular volume, MCH: mean corpuscular hemoglobin, MCHC: mean corpuscular hemoglobin concentration). (*E*) Effect on blood counts. The p-values for panels (*B*–*E*) were determined by one-way ANOVA. All p-values are not corrected for multiple hypothesis testing.

In prokaryotes, dCTP deamination to dUTP serves as a mechanism of defense against phage infections (26), suggesting a possible role of CDADC1 in antiviral defense. To test this hypothesis, we queried OncoDB (27), which summarizes data from The Cancer Genome Atlas (28) for possible upregulation of CDADC1 in response to viral infection in various types of malignancies (Fig. 5*A*). In most cancer types, there was no upregulation in response to infection with different viruses. Moderate upregulation of CDADC1 in response to virus infection was only observed in some adenocarcinomas (Fig. 5*A* and *SI Appendix*, Fig. S23), but was not significant.

#### No synthetic lethality of Cdadc1 with Dctd.

To test for a possible physiological role of CDADC1, we created a mouse model (in the C57BL/6JRj genetic background) with inactivation of *Cdadc1*, by mutation of the catalytic glutamate residue (E401 in the mouse) to alanine, using ssDNA template-directed repair of a Cas9 induced double-strand break (*SI Appendix*, Fig. S24). The resulting mice did not exhibit any obvious phenotypes, and both males and females were fertile. This finding suggested to us that CDADC1/Cdadc1 may have a physiologically redundant role with DCTD/Dctd, despite acting at the level of the tri- rather than the monophosphates. To test this hypothesis, we created a *Cdadc1/Dctd* double KO (dKO) mouse, by coinjecting sgRNA guides for *Cdadc1* and *Dctd*. For *Cdadc1*, the sgRNA as for the active site point mutation was reused, but without the repair template. For *Dctd*, we used a pair of sgRNAs on either side of the third coding exon (fourth exon of the transcript). The initial founder mouse had a 17 bp deletion in *Cdadc1* (*SI Appendix*, Fig. S25) and the intended 281 bp deletion, with 115 bp of the fragment inserted in an inverted orientation, in *Dctd*, causing a Tyr396fs*7 frameshift mutation (*SI Appendix*, Fig. S26). Crossing of mice with monoallelic *Cdadc1* and *Dctd* KO created offspring without drastic deviations from Mendelian genetics [*SI Appendix*, Table S2; equally large or larger deviations from Mendelian genetics expected with ~40% probability according to the χ^2^ test (29)]. In particular, the offspring included 2 homozygous dKO mice. The dKO mice had no overt phenotype (*SI Appendix*, Fig. S27), and a female dKO mouse was fertile. This was surprising because the *Dctd* KO alone should cause male and female infertility according to the International Mouse Phenotyping Consortium (30). In our hands, however, the *Dctd* KO was both male and female fertile, which is consistent with the low *Dctd* mRNA read counts in the testis (31).

Next, we analyzed five double heterozygous mice (henceforth termed WT mice), nine *Cdadc1* KO mice (henceforth termed CKO mice) and six *Cdadc1/Dctd* double KO mice (henceforth termed dKO mice) in more detail. One dKO mouse had a very impaired gait, which could be a sporadic phenotype, or an unrelated problem. The other mice appeared normal, and indistinguishable from wild-type mice.

First, we compared the overall body mass and individual organ masses for the females that represented the majority in our cohort. The data did not reveal systematic differences in overall body mass, or the masses of prominent organs, with the possible exception of the spleen (before, but not after multiple hypothesis correction) (Fig. 5*B*). When males were included in the analysis and the organ masses were normalized with respect to body mass, relative spleen mass was not significantly altered (*SI Appendix*, Fig. S28). Metabolic indicators were also similar in the three groups. Specifically, Alanine Transaminase and Aspartate Transaminase levels provided no indication of liver pathologies, and an increase in glucose levels of CKO and dKO mice compared to WT was not significant (Fig. 5*C*). Red blood cell parameters were analyzed in females and also showed no signs of perturbation in CKO and dKO mice. In particular, RBC count, hemoglobin, hematocrit (red blood cell volume/total volume of blood), mean corpuscular volume (MCV, a measure of the average size of red blood cells), mean corpuscular hemoglobin (MCH, the average amount of hemoglobin per red cell, used to detect anemia), and mean corpuscular hemoglobin concentration (MCHC, amount of hemoglobin per red blood cell) were all similar in all three groups (Fig. 5*D*). Finally, blood cell counts were also similar. In particular, differences in the total amount of leukocytes, neutrophils, lymphocytes, monocytes, and eosinophils were all not significant. For erythroblasts, differences were significant only before, but not after correction for multiple hypothesis testing (Fig. 5*E*).

## Discussion

In this work, we provide biochemical and structural evidence that human CDADC1 deaminates dCTP and CTP (Fig. 1). CDADC1 structures in the absence of the ligand, and in the presence of dCTP and ^5m^dCTP reveal both the molecular details of its substrate specificity and the mechanism of regulation (Figs. 2–4). CDADC1 consists of an inactive N-terminal domain, with an architectural role, and of an active C-terminal domain, which contains the deaminase active site (Fig. 2*A*). The C-terminal domain features an unusually narrow pocket for the base that selects for unmodified cytosine, and a highly cationic pocket for the triphosphate of dCTP (Fig. 3). Substrate-induced structural changes and the catalytic activity of CDADC1 are intricately intertwined. In the absence of a substrate, CDADC1 can exist as either a trimer or a relaxed hexamer. Substrate binding induces a cascade of conformational changes that ultimately lead to the formation of a tight hexamer, the active form of CDADC1 (Fig. 4). The biochemically observed cooperativity of the deamination reaction (Fig. 1*F*) is likely due to the cross-talk between active sites, and not due to the binding of substrate to the putative allosteric site in the C-terminal domain, which does not appear to be used, at least not in a way similar to DCTD.

The physiological role of the CDADC1 is still enigmatic. In vitro, the best substrate for CDADC1 is dCTP, but the preference over CTP is only ~twofold. As CTP concentration in cells (40 to 520 μM) is much higher than that of dCTP (10 to 50 μM) (32), the preference of CDADC1 for dCTP may be overwhelmed in vivo by substrate abundances. At present, we cannot even exclude that there may be yet another, still unidentified physiological substrate. Assuming that the physiological substrate of CDADC1 is dCTP, it is possible that CDADC1 may simply serve as a defense against mutagenic imbalances in the 2′-deoxynucleotide pool, by guarding against excess dCTP. However, the K_d_ or K_half_ in the region of 200 μM would have to be reconciled with the considerably lower reported dCTP concentrations (32). Alternatively, CDADC1 may have a role similar to the role of dCTP deaminases in prokaryotic/phage biology.

We considered the possibility that CDADC1 may have antiviral function. Similar to prokaryotic dCTP deaminases in antiphage defense (26), CDADC1 may be activated/overexpressed in response to viral infection. The enzyme may then overload the replicative polymerase with dUTP, causing excess dU in genomic DNA, single-strand breaks from UDG activity, and ultimately cell death (33). However, CDADC1 is not significantly overexpressed in human cell lines in response to (chronic) viral infection (Fig. 5*A*). The result speaks against the immune hypothesis, but does not rule it out completely, since only chronic infection was tested (Fig. 5*A*).

We also considered the possibility that CDADC1 may play a metabolic role. dTTP is synthesized from dUMP. Prior to our work, there were already several routes leading to dUMP: a) from dU, catalyzed by thymidine kinase (salvage pathway) (34), b) from UDP, catalyzed by ribonucleotide reductase, a kinase and a triphosphatase (35–37), and c) from dCMP, catalyzed by DCTD (38). The combination of the dCTP deaminase activity of CDADC1 and dUTP triphosphatase (DUT) activity could add yet another pathway. According to this model, we would not expect a strong phenotype for the mouse *Cdadc1* KO, but there might be a stronger phenotype for the *Cdadc1/Dctd* dKO. The lack of an obvious phenotype for the dKO (Fig. 5 *B*–*E*) does not support this model. However, it remains possible that the CDADC1 pathway may still be required as a backup, in case more than just the dCMP pathway to dUMP is compromised.

In summary, our data show that the vertebrate protein CDADC1 has (d)CTP deaminase activity and provide its structural background. However, why an enzyme with this activity would have evolved in vertebrates remains to be investigated.

## Materials and Methods

For complete Materials and Methods, see the *SI Appendix*, *Materials and Methods*.

### CDADC1 Expression.

CDADC1 and its E400A variant were expressed from codon-optimized genes (GeneArt, Thermo Fisher Scientific) with N-terminal, thrombin-cleavable hexahistidine tag (MGSSHHHHHHSSGLVPRGSH) in *E. coli* BL21-CodonPlus (DE3)-RIL cells. The proteins were then purified by affinity chromatography (Cytiva His-trap and Heparin HP columns), and sizing chromatography (Superdex 200 Increase 10/300 GL column).

### Measurement of CDADC1 Activity Using UHPLC or Ammonia Detection.

CDADC1 activity was monitored by resolving (2′-deoxy)nucleotides on an ACQUITY UHPLC HSS T3 Column, 100 Å, 1.8 µm, 2.1 mm × 150 mm at 0.3 mL/min operated by the ACQUITY UHPLC System (Waters). Samples were resolved using the following gradient profile: 0 to 1.5 min. 1% B, 1.5 to 11.5 min 1% to 10% B, 19 to 24 min 10% to 100% B, where A was 20 mM ammonium formate pH 4.4 and B methanol. Alternatively, the release of ammonia was quantified using an Ammonia Assay Kit (MAK310, Sigma-Aldrich). Kinetic data were analyzed using GraphPad Prism.

### MP.

MP experiments were performed with a Refeyn TwoMP mass photometer (Refeyn Ltd.) using AcquireMP software (Refeyn Ltd.).

### Cryo-EM.

Samples were blotted on glow-discharged Holey carbon grids (Quantifoil R 1.2/1.3, copper, mesh 200). Cryo-EM data were collected on Talos Arctica G2 (Thermo Fisher Scientific) operated at 200 kV, equipped with an X-FEG electron source, BioQuantum imaging filter (Gatan), and K3 direct electron detector (Gatan). Imaging was performed in counting mode at a nominal magnification of 100,000x (pixel size of 0.81 Å/pixel), with a dose of ~40 e^−^/Å^2^ and a defocus range between 0.8 to 2.2 μm (Apo) and or 0.8 to 2.5 μm (dCTP and ^5m^dCTP complexes). Data were analyzed using CryoSPARC, using protocols for heterogeneous refinement. For model building, AlphaFold2 (24) generated CDADC1 monomers were rigid body fitted into the map using UCSF ChimeraX v1.4 (39) and manually adjusted using Coot (40). Phenix 1.20.1 was used for automated real-space refinement (41).

### Generation of Mouse Lines.

Animal experiments were approved by the II Local Ethical Committee in Warsaw affiliated with the Warsaw University of Life Sciences (approval number WAW2/011/2022) and were performed according to Polish Law (Act number 266/15.01.2015). Female mice were superovulated (10 IU of Pregnant Mare Serum Gonadotropin, Folligon, Intervet, Netherlands, ~50 h later 10 IU of Human Chorionic Gonadotropin; Chorulon, Intervet, Netherlands), and mated immediately after the second injection. 21 to 22 h later, zygotes were microinjected with Cas9-gRNA mixes for *Cdadc1* or *Dctd* KO and Cas9-gRNA-repair template for the *Cdadc1* E401A (E401A in mouse corresponds to E400A in human) mutation and surgically transferred to pseudopregnant females. For genotyping, DNA was isolated from ear or toe fragments (surplus tissue from animal identification).

## Supplementary Material

Appendix 01 (PDF)

## Data Availability

Models and cryo-EM maps data have been deposited in PDB and EMDB [9HFQ (42), 9HFR (43), 9HFS (44), and 9HFT (45), EMD-52123 (46), EMD-52125 (47)].
