# Supplementary material for: Activity and structure of human (d)CTP deaminase CDADC1
Source: Proc Natl Acad Sci U S A. 2025 May 5;122(19):e2424245122. doi: 10.1073/pnas.2424245122 (PMC12088426; doi:10.1073/pnas.2424245122)
Supplement: Supplementary file 1 — Appendix 01 (PDF) [file pnas.2424245122.sapp.pdf]

PNAS

Supporting Information for

Activity and structure of human (d)CTP deaminase CDADC1

Anton Slyvka [1*], Ishan Rathore [2], Renbin Yang [3], Olga Gewartowska [1], Tapan Kanai [3], George T. Lountos [4], Krzysztof Skowronek [1], Mariusz Czarnocki-Cieciura [1], Alexander Wlodawer [2*], Matthias Bochtler [1,5*]

1. International institite of Molecular and Cell Biology in Warsaw, Trojdena 4, 02-109 Warsaw, Poland
2. Cancer Research Technology Program, Leidos Biomedical Research Inc., Frederick, MD 21701, USA.
3. Center for Structural Biology, National Cancer Institute, NIH, Frederick, MD 21702, USA
4. Basic Science Program, Frederick National Laboratory for Cancer Research, Frederick, MD, 21702, USA
5. Institute of Biochemistry and Biophysics, Pawinskiego 5a, 02-106 Warsaw, Poland

* Anton Slyvka, Alexander Wlodawer, Matthias Bochtler
Email: aslyvka@iimcb.gov.pl, wlodawer@nih.gov, mbochtler@iimcb.gov.pl

This PDF file includes:

Supporting text
Figures S1 to S28
Tables S1 to S2
SI References

Supporting text

Materials and Methods

Molecular cloning and site-directed mutagenesis

Open reading frame of human CDADC1 (UniProt ID: Q9BWV3; 514 amino acids), codon-optimized for expression in *E. coli*, was ordered from GeneArt (Thermo Fisher Scientific). It was inserted under the T7 promoter into pET28a expression vector using NdeI and XhoI restriction sites. The resulting construct encoded the protein that contained the N-terminal 6xHis tag and Thrombin cleavage site (MGSSHHHHHHSSGLVPRGSH) and consisted of total 534 amino acids (molecular weight 60.618 kDa). The E400A variant of CDADC1 was generated by PCR followed by degradation of template DNA by DpnI. The following primers were used for site-directed mutagenesis: FW 5' CCGCTATATTATCCATGCAGcACAGAATGCACTGACCTTTCG 3', RV 5' CGAAAGGTCAGTGCATTCTGTgCTGCATGGATAATATAGCGG 3'. *E. coli* Top10 strain (Invitrogen) was used for plasmid amplification.

CDADC1 expression

Both wild-type and E400A variants of human CDADC1 were expressed in *E. coli* BL21-CodonPlus (DE3)-RIL strain. The starter culture inoculated from freshly transformed cells was grown overnight in LB media supplemented with 50 µg/ml kanamycin (Kan), 34 µg/ml chloramphenicol (Cm), and 1% glucose at 37 °C with shaking at 140 rpm. For expression, the starter culture was diluted 1:200 with the fresh LB media (Kan, Cm, no glucose) and grown at 37 °C, 140 rpm till OD600 reached 0.6. The culture was then chilled to approximately 10 °C, supplemented with 0.25 mM IPTG, and grown at 25 °C, 140 rpm for 16 h. The cells were collected by centrifugation at 4000g for 20 min, washed with PBS (pH 7.4) and stored at -20 °C.

CDADC1 purification:

All protein purification steps were carried out at the lowest practicable temperature (0 – 12 °C) using filtered (0.45 µm) and degassed buffers. The equipment used for purification was ÄKTA Purifier FPLC system (GE Healthcare) and Minipuls 3 peristaltic pump (Gilson). The progress of purification was monitored by the UV 280/254 absorbance and by the SDS-PAGE electrophoresis. Pellets of CDADC1 expressing cells, typically from 4-6 liters of culture, were thawed on ice, suspended in Buffer 1 (50 mM Sodium Phosphate, pH 7.0, 750 mM NaCl, 20% glycerol, 2 mM imidazole, 10 mM 2-mercaptoethanol (2-ME), and 1 mM phenylmethylsulfonyl fluoride (PMSF)) and lysed by sonication. The lysates were clarified by centrifugation at 15000g for 40 min. The soluble fraction was applied on 5 mL HisTrap HP column (Cytiva) equilibrated with Buffer 1. The column was washed with 100 ml of each: Buffer 2 (50 mM Sodium Phosphate, pH 7.0, 2000 mM NaCl, 20% glycerol, 2 mM imidazole, 10 mM 2-ME), Buffer 3 (50 mM Sodium Phosphate, pH 7.0, 750 mM NaCl, 20% glycerol, 20 mM imidazole, 10 mM 2-ME), Buffer 4 (20 mM Tris-HCl, pH 7.0, 500 mM NaCl, 10% glycerol, 2 mM imidazole, 10 mM 2-ME) at 2 ml/min flow rate. The protein was eluted by 5% step gradient between Buffer 4 and Buffer 5 (20 mM Tris-HCl, pH 7.0, 500 mM NaCl, 10% glycerol, 1000 mM imidazole, 10 mM 2-ME) at 1 ml/min flow rate. The majority of CDADC1 usually eluted at 15% Buffer 5 (~150 mM imidazole). The highest purity CDADC1 fractions were diluted with Buffer 6 (20 mM Tris-HCl, pH 7.0, 5 mM NaCl, 10% glycerol, 10 mM 2-ME) in 1:4 ratio and applied on 5 mL HiTrap Heparin HP column (Cytiva) equilibrated with Buffer 7 (20 mM Tris-HCl, pH 7.0, 100 mM NaCl, 10% glycerol, 10 mM 2-ME). The column was washed with 100 ml of Buffer 7 at 2 ml/min flow rate. The protein was eluted with 60 ml linear gradient between Buffer 7 and Buffer 8 (20 mM Tris-HCl, pH 7.0, 1000 mM NaCl, 10% glycerol, 10 mM 2-ME) at 1 ml/min flow rate. The best CDADC1 fractions were concentrated and their buffer was exchanged to Buffer 9 (1x PBS, pH 7.4, 1000 mM NaCl, 10 mM 2-ME)* using diafiltration in the Amicon 30 kDa MWCO filter units (Merck). The 0.5 ml of CDADC1 sample was resolved on Superdex 200 Increase 10/300 GL column (Cytiva). The most homogeneous fractions were buffer exchanged to Buffer 10 (2x PBS, pH 7.4, 10 mM 2-ME), concentrated to 0.5 ml and re-applied on Superdex column equilibrated with Buffer 10. The best CDADC1 fractions were flash-frozen in liquid nitrogen and stored at -80 °C or mixed 1:1 with

glycerol and stored at -20 °C. PBS was prepared according to the standard recipe, where 1x PBS contains 10 mM Na_2HPO_4, 1.8 mM KH_2PO_4, 137 mM NaCl and 2.7 mM KCl (pH 7.4).

Analytic size-exclusion chromatography

The oligomeric state of CDADC1 protein was tested using a Superdex 200 Increase 10/300 GL column and ÄKTA Purifier FPLC system. The sample containing 0.1 mg of CDADC1 wild-type in 0.5 mL of Buffer 10 was applied on the column pre-equilibrated with the same buffer and resolved at 0.4 mL/min flow rate. Gel Filtration Standard (Bio-Rad) resolved at the same conditions was used to generate a standard curve.

Measurement of CDADC1 activity using UHPLC

Prior to any biochemical assay the concentration of each tested substrate (nucleobases, (2'-deoxy) nucleosides, (2'-deoxy)nucleotide monophosphates/triphosphates) was independently determined using UV spectroscopy at the wavelength corresponding to its A_{max} and using publicly available extinction coefficients. 0.1 µM of CDADC1 (monomer) was mixed with 1000 µM of tested substrate in the Reaction Buffer (1.3x PBS (pH 7.4), 1 mM zinc acetate, and 10 mM 2-ME). The reaction was incubated at 37 °C for 2 h followed by the treatment with digestion/dephosphorylation mix (1). The resulting ribo- and deoxyribonucleosides and bases were resolved on the ACQUITY UHPLC HSS T3 Column, 100 Å, 1.8 µm, 2.1 mm X 150 mm at 0.3 ml/min operated by the ACQUITY UHPLC System (Waters). Samples were resolved using following gradient profile: 0 – 1.5 min. 1% B, 1.5 – 11.5 min 1% to 10% B, 19 – 24 min 10% to 100% B, where A was 20 mM ammonium formate pH 4.4 and B – methanol. Absorbance was measured at 260 nm. The results were analyzed using the Empower 3 software (Waters).

CDADC1 activity assay: ammonia detection method

In order to measure the deaminase activity of CDADC1 in both qualitative and quantitative ways the Ammonia Assay Kit (MAK310, Sigma-Aldrich) was used according to the manufacturer instructions. At the early stages of CDADC1 biochemical characterization it was established that the working solution of the kit fully inactivates CDADC1, which allowed for the direct and accurate measurement of its activity. At each time point, 10 µl aliquot was mixed with 90 µl of the assay working solution that was pre-pipetted into the 96-well black assay plate (Corning, REF3991).

For comparison of CDADC1 activity on different substrates (Fig. 1B), 0.5 µM of CDADC1 (monomer) was mixed with 1 mM of tested substrate in the High Salt Reaction Buffer (1x PBS pH 7.4, 12 mM NaCl, 140 mM KCl, 1 mM $MgCl_2$, 1 mM Zn Acetate, 10 mM 2-ME) and incubated at 37 °C. Aliquots were taken at 0, 2, 5, 10, 20, 30, 40, 50 and 60 min. The fluorescence was measured using Tecan Infinite M1000 plate reader at the following settings: λ_{ex} 360 nm, λ_{em} 450 nm, bandwidth 5 nm, gain 50, number of flashes 100, flash frequency 400 Hz.

For testing of the allosteric regulation of CDADC1 activity on dCMP (Fig. S5AB), 0.2 µM of CDADC1 (monomer) was mixed with 1 mM dCMP in the presence of 50 µM of dCTP or dTTP in the Reaction Buffer that either contained 2 mM $MgCl_2$, or did not. Aliquots were taken at 0, 5, 10, 15, 20, 25 min. The fluorescence was measured using Tecan Infinite M1000 plate reader at the following settings: λ_{ex} 360 nm, λ_{em} 450 nm, bandwidth 5 nm, gain 100, number of flashes 100, flash frequency 400 Hz.

For testing the inhibition of CDADC1 activity on dCTP (Fig. S5C), 0.5 µM of CDADC1 (monomer) was mixed with 1 mM dCTP in the High Salt Reaction Buffer (1x PBS pH 7.4, 12 mM NaCl, 140 mM KCl, 1 mM $MgCl_2$, 1 mM Zn acetate, 10 mM 2-ME) in the presence of increasing concentrations (0.1, 0.5, 10, 100, and 1000 µM) of dTTP, dTMP, UTP, and CMP and incubated for 1 h. The fluorescence was measured using Tecan Infinite M1000 plate reader at the following settings: λ_{ex} 360 nm, λ_{em} 450 nm, bandwidth 5 nm, gain 50, number of flashes 50, flash frequency 400 Hz.

CDADC1 steady-state kinetics

For determining the kinetic constants of CDADC1, ammonia detection method was utilized. We focused on three substrates for which CDADC1 had significant activity: dCTP, CTP, and dCMP. Firstly, we determined the dynamic range of CDADC1 concentration for dCTP, CTP, and dCMP by carrying out the reaction in the presence of 0.1 - 0.5 μM CDADC1 (monomer) and 5000 μM substrate. For subsequent Michaelis-Menten kinetics, the protein concentration, substrate concentration range, and time points were carefully selected for each substrate, so that the produced ammonia stays within linear detection range (12 – 1000 μM) of the assay. Next step was to determine the substrate concentration at which CDADC1 initial velocity reaches the plateau. Final useful parameters were the following: dCTP (50 – 1000 μM dCTP, 0.2 μM CDADC1, 0 – 10 min, time points every 2 min), CTP (100 – 2500 μM CTP, 0.3 μM CDADC1, 0 – 10 min, time points every 2 min), dCMP (500 – 20000 μM dCMP, 0.3 μM CDADC1, 0 – 25 min, time points every 5 min). Concentrations of the reaction products were plotted against the time and the initial velocities (μM/min) at each substrate concentration were determined from the linear portions of the graphs. Initial velocities from at least three biological repeats were plotted against the substrate concentration (μM) in GraphPad Prism V10. The data were analyzed using Prism nonlinear regression fits for both Michaelis-Menten ($Y = V_{max}*X/(K_M + X)$) and Allosteric sigmoidal ($Y=V_{max}*X$^h/(K_{half}^h + X^h)) models, where X is a substrate concentration, Y is an initial velocity (V_0), h is a Hill coefficient, V_{max} is maximum velocity, K_M is Michaelis-Menten constant (substrate concentration for which $V_0 = 1/2V_{max}$), and K_{half} is an equivalent of K_M in the sigmoidal model. Catalytic constants (k_{cat}, min^{-1}) were calculated by dividing the V_{max} (μM/min) by CDADC1 concentration (μM).

Test of the phosphatase activity of CDADC1

In order to test if CDADC1 has phosphatase activity in addition to its deaminase activity, we utilized the different strength of binding of mono- or triphosphorylated (2'-deoxy)nucleotides to the anion exchange resins. 500 μM of dCTP was mixed with 1 μM CDADC1 in 20 μl of the Reaction Buffer and incubated for 1 h at 37 °C, followed by overnight incubation at room temperature. The samples were than mixed with 4980 μl of Q1 Buffer (20 mM Tris-HCl, pH 8.0) applied on 5 ml HiTrap Q HP anion exchange column (Cytiva) equilibrated with Q1 buffer at 1 ml/min flowrate. The column was washed with 10 ml of Q1 buffer, and the sample was eluted by linear gradient (0 – 70%) between Q1 and Q2 (20 mM Tris-HCl, pH 8.0, 500 mM NaCl) buffers at 2 ml/min. The absorbance was collected at two wavelengths, corresponding to A_{max} of cytosine (271 nm) and uracil (262 nm). dCMP was used as a reference of the weaker binding to Q-sepharose.

Mass photometry

Mass photometry experiments were performed with Refeyn TwoMP mass photometer (Refeyn Ltd.) using AcquireMP software (Refeyn Ltd.). Before the measurement, a self-adhesive 6-well silicone sample template was placed on the microscope slide (24 x 50 mm, No. 1.5H) that was thoroughly washed with water and isopropanol and dried with nitrogen. The instrument was calibrated using NativeMark Protein Standard (Thermo Fisher Scientific). A drop of 16 μl of CDADC1 E400A variant at 0.063 μM (monomer) in Buffer 10 was used in each measurement. To capture CDADC1 oligomerization dynamics, the final dilution was made directly in the drop (1 μl of 1 μM + 15 μl Buffer 10) and recorded every 5 minutes for 15 minutes. To evaluate the effects of substrate binding on CDADC1 oligomerization dynamics 1 mM of dCTP was added, either directly to the drop, or pre-incubated with CDADC1 at room temperature for 10 minutes. MP signals were recorded for 60 s at room temperature. The data was analyzed using the DiscoverMP software.

Negative stain electron microscopy

300-mesh cooper grid (TED PELLA 01843-F) was glow discharged at 20 mA for 60 seconds using Q150T ES instrument (Quorum Technologies). 2 μl 0.22 μM CDADC1 wt (monomer) was applied to a grid and stained with 1% uranyl acetate. TEM images were acquired with Tecnai T12 BioTWIN 120 kV Electron Microscope (FEI) at 49000x magnification.

Cryo-EM grid preparation and data collection

Cryo-EM structures of three CDADC1 E400A samples were determined: CDADC1 alone (Apo), in complex with dCTP (C-dCTP), and with ^{5m}dCTP (C-^{5m}dCTP), respectively. Samples were prepared in Buffer 10 at a final concentration of 1 mg/ml (Apo), 0.7 mg/ml (with C-dCTP), and 0.8 mg/ml (with C-^{5m}dCTP). C-dCTP and C-^{5m}dCTP were supplemented at 1 mM, and the samples were kept on ice for at least 30 minutes. Holey carbon grids (Quantifoil R 1.2/1.3, copper, mesh 200) were glow discharged at 15 mA for 25 seconds (Apo and C-dCTP) or for 5 seconds (C-^{5m}dCTP) using PELCO easiGlow System immediately before sample loading. 3 µl of sample was applied on grid placed in a Vitrobot Mark IV (Thermo Fisher Scientific) under 100% humidity at 4 °C. Under these conditions, different parameters were tested, and the grids that turned out to be suitable for data collection were prepared as follows: Apo and C-^{5m}dCTP (wait time 5 s, blot 2 s, force 0, drain 0), C-dCTP (wait time 10 s, blot 4 s, force 0, drain 0). Grids were then plunged into liquid ethane and stored in liquid nitrogen. Cryo-EM data was collected on Talos Arctica G2 (Thermo Fisher Scientific) operated at 200 kV, equipped with an X-FEG electron source, BioQuantum imaging filter (Gatan) and K3 direct electron detector (Gatan). Imaging was performed in counting mode at a nominal magnification of 100,000x, corresponding to a pixel size of 0.81 Å/pixel. Movies were collected at a dose rate of 11.5 e^-/px/s (44 e^-/Å^2) (Apo), 11.2 e^-/px/s (42.6 e^-/Å^2) (dCTP) and 11.3 e^-/px/s (42.9 e^-/Å^2) (^{5m}dCTP), exposure time of 2 s, at a defocus range from 0.8 – 2.2 µm (Apo) and from 0.8 – 2.5 µm (dCTP and ^{5m}dCTP). Data was collected using EPU software (Thermo Fisher Scientific).

Cryo-EM data processing:

The general data processing workflow used for all datasets is described here, and further details are provided in Figure S10. Movie frames were motion-corrected, dose-weighted, and down-sampled by a factor of two using Fourier cropping by patch motion correction job. Further CTF correction was applied with patch CTF correction job in CryoSPARC (2). Particles were manually picked to generate templates for subsequent template picking. Template-picked particles were extracted with a box size of 256 pixels and multiple rounds of 2D classification were performed to remove junk. Cleaned particle stacks were then used for *ab initio* reconstruction with four classes. These classes were then used in a subsequent heterogeneous refinement job to separate the trimeric and hexameric complexes. One hexamer, one trimer, and two junk classes were used during heterogeneous refinement to remove junk particles. The classes were then refined using Non-Uniform (NU) refinement (3) and further local refinement with and without applying symmetry. The resulting maps were evaluated, and the resolution was determined for a Fourier Shell Correlation (FSC) of 0.143.

Model building and refinement

A model of human CDADC1 monomer was generated using AlphaFold2 (4). Six copies of CDADC1 monomer were rigid body fitted into the maps using the fit in map function of the UCSF ChimeraX v1.4 (5). Coot 0.9.1 was used for manual real-space refinement and building (6). Phenix1.20.1 was used for automated real-space refinement (7). Grade Web Server (https://grade.globalphasing.org) (8) was used for building of ^{5m}dCTP ligand. CDADC1 models were validated using MolProbity within Phenix. Figures were prepared using ChimeraX. Cryo-EM data, maps, and models were submitted to EMDB and PDB (Table S1).

Generation of mouse lines

Animal experiments were approved by the II Local Ethical Committee in Warsaw affiliated to the Warsaw University of Life Sciences (approval number WAW2/011/2022) and were performed according to Polish Law (Act number 266/15.01.2015).

The *Cdacd1*^{Glu401Ala} catalytic mutant mouse line was generated in C57BL/6JRj genetic background using the CRISPR/Cas9 method. In addition to single nucleotide substitution causing Glu401Ala mutation, a silent mutation in Ala400 was introduced in order to destroy the PAM sequence once the desired mutation is introduced.

The CRISPR cocktail consisted of mRNA Cas9 (25 ng/µl, Thermo Fisher Scientific), sgRNA (15 ng/µl; Thermo Fisher Scientific) and dsDNA repair template (6 ng/µl). DNA sequence corresponding to the variable region of the gRNA and [PAM]: GTTCAGATACATCATACATG[CGG]; ssDNA repair template sequence:
TATGCCGACTTCCCGCACATGGATGACAAGCATAAGGACAGAGAAATACGGAAGTTCAGATACATCA TACATGCaGcGCAGAACGCTTTGACATTTAGgtaagagctgtttttagcctctgtcctttaaaagacaacagaggatggttgag.
Donor mice were injected first with 10 IU of PMSG (Pregnant Mare Serum Gonadotropin; Folligon, Intervet, Netherlands) and ~50 hours later with 10 IU of hCG (Human Chorionic Gonadotropin; Chorulon, Intervet, Netherlands) to induce superovulation. Females were mated with males immediately after hCG injection. Zygotes were collected from mated females 21–22 h post hCG injection. Zygotes, were microinjected into the cytoplasm using Eppendorf 5242 microinjector (Eppendorf-Netheler-Hinz GmbH) and Eppendorf Femtotips II capillaries and 24 h post microinjection surgically transferred to pseudo-pregnant females. Pups were genotyped at around 4 weeks. The presence of mutation was confirmed by sequencing in the founder mouse and, after backcrossing, in N1 generation mice.

The *Cdadc1/Dctd* dKO mouse line was generated similarly, by co-injection of sgRNA targeting *Cdadc1* and *Dctd*. For *Cdadc1*, the same guide as for the generation of the catalytic mutant was used, but without the repair template. For Dctd, a pair of gRNAs targeting introns surrounding exon 4 of *Dctd* (DNA sequence corresponding to the variable region of the gRNA and [PAM]: CAGCGGCTAACTCCCGAGTG [AGG] for gRNA1 and ATGCAGACGTGACAAATCCA [GGG] for gRNA2). As a result, a del4bp, del17bp, and del23bp mutations were introduced in *Cdadc1* and an ins115;del281bp change causing the deletion of exon 4 was introduced in *Dctd*.

Mouse Genotyping

Genomic DNA was isolated from ear or toe fragments (surplus tissue from animal identification) using the HotSHOT method (9): the tissue fragments were incubated for 25 minutes in 100 µl of lysis buffer (25 mM NaOH, 0.2 mM EDTA) at 95 °C. After cooling down to room temperature, 100 µl of neutralization buffer (40 mM Tris-HCl; pH 5) was added. The solution containing genomic DNA was stored at 4 °C. PCR was run using the Phusion High-Fidelity DNA Polymerase (Thermo Fisher Scientific, F-530S) according to the manufacturer’s instructions. Genotyping primers were TGAGACTCACTGTGCTTCTGC (Fw) and TGGGCAAGTCCCTAAGGAC (Rv) for CDADC1 and AACAAACCAAGGCCAGCTG (Fw) and GAAGATCAAACCCGCCTGC (Rv) for DCTD. Additionally, for Cdadc1^{Glu401Ala} and Cdadc1 KO alleles, restriction digestion was performed, using HhaI or XceI (Thermo Fisher Scientific) enzymes, respectively.

Supplementary Tables

PDB / EMDB	CDADC1 Apo: Trimer<br>9HFQ / EMD-52121	CDADC1 Apo: Hexamer<br>9HFR / EMD-52122	CDADC1 + dCTP: Trimer<br>- / EMD-52123	CDADC1 + dCTP: Hexamer<br>9HFS / EMD-52124	CDADC1 + ^{5m}dCTP: Trimer<br>- / EMD-52125	CDADC1 + ^{5m}dCTP: Hexamer<br>9HFT / EMD-52126
**Data collection**						
Magnification	100,000	100,000	100,000	100,000	100,000	100,000
Voltage (kV)	200	200	200	200	200	200
Electron exposure ($e^-/Å^2$)	44	44	42.6	42.6	42.9	42.9
Defocus range (μm)	-2.2 – -0.8	-2.2 – -0.8	-2.5 – -0.8	-2.5 – -0.8	-2.5 – -0.8	-2.5 – -0.8
Pixel size (Å)	0.81	0.81	0.81	0.81	0.81	0.81
Micrographs collected (no.)	5981	5981	5321	5321	6329	6329
**Data processing**						
Total extracted particles (no.)	5.2M	5.2M	5.7M	5.7M	7.3M	7.3M
Particle used for 3D (no.)	605,350	605,350	2,031,349	2,031,349	2,793,395	2,793,395
Final particles (no.)	162,719	20,517	425,492	310,460	885,243	112,912
Symmetry imposed	C3	D3	C3	D3	C3	D3
**Map resolution (Å), FSC: 0.143**						
EMDB FSC server	3.06	3.7	3.08	2.8	2.81	2.9
Mtriage (masked/unmasked)	2.6/2.7	3.1/3.2	2.7/2.8	2.4/2.4	2.5/2.5	2.4/2.6
Map resolution range (Å)	2.505 - 8.685	3.05 - 11.55	2.531 - 9.045	2.27 – 8.78	2.285 - 8.638	2.38 – 8.87
**Refinement**						
Model resolution (Å), FSC: 0.5	2.7	3.2		2.4		2.6
Model composition						
Polymer chains	3	6		6		6
Non-hydrogen atoms	9132	18204		18510		18516
Protein residues	1155	2298		2358		2358
Ligands	ZN: 6	ZN: 12		ZN:12, DCP (dCTP): 6		ZN: 12, A1I2I (^{5m}dCTP): 6
Water	3	6		6		6
Occupancy = 1 (%)	100	100		100		100
**B factors**						
Iso/Aniso (#)	9132/0	18204/0		18510/0		18516/0
Protein (min/max/mean)	0.00/89.26/24.46	0.00/69.47/30.81		0.00/56.74/19.40		0.00/59.95/10.39
Ligand (min/max/mean)	73.96/124.82/96.36	53.59/92.70/77.71		22.55/57.80/31.69		10.26/66.97/25.33
Water (min/max/mean)	1.64/6.36/4.58	25.80/33.16/28.79		5.34/10.03/6.88		0.00/5.34/1.82
**Validation**						
**Root-mean-square deviations**						
Bond lengths (Å)	0.002	0.002		0.002		0.003
Bond angles (°)	0.45	0.409		0.479		0.484
MolProbity score	1.32	1.19		1.27		1.28
Clashscore	5.79	4.03		5.07		5.24
Rotamer outliers (%)	0	0		0		0
**Ramachandran plot**						
Favored (%)	98.03	98.47		98.13		98.65
Allowed (%)	1.97	1.53		1.87		1.35
Disallowed (%)	0	0		0		0
**Model to Map fit (CC)**						
mask	0.88	0.89		0.89		0.88
box	0.74	0.76		0.76		0.76
peaks	0.72	0.73		0.76		0.74
volume	0.82	0.85		0.84		0.83
mean ligand	0.78	0.84		0.84		0.80

Table S1. Cryo-EM data collection, data processing, and model building. Data were collected in the absence of ligand, in the presence of dCTP, and in the presence of ^{5m}dCTP. All data were processed imposing either C3 symmetry ("trimer") or D3 symmetry ("hexamer"). Irrespective of whether the ligands were present in solution or not, trimers did not contain them. By contrast, the ligands that were present in solution were observed to be bound to some hexamers. Therefore, altogether four models were submitted to the PDB, a trimer without ligand, a hexamer without ligand, a hexamer with bound dCTP, and a hexamer with bound ^{5m}dCTP.

Total: 32 pups		*Dctd*		
		homozygous	heterozygous	WT
Cdadc1	homozygous	2 (2)	5 (4)	1 (2)
	heterozygous	8 (4)	5 (8)	4 (4)
	WT	0 (2)	4 (4)	3 (2)

Table S2. Offspring of a cross of mice with mono-allelic *Dctd* and *Cdadc1* mutations. Altogether 24 pups were obtained and genotyped. The table indicates the genotypes of the offspring, and the observed numbers of offspring. Numbers in brackets indicate the expected numbers, based on Mendelian inheritance, since *Dctd* and *Cdadc1* are located on different chromosomes in mice (*Dctd* is on chromosome 8, *Cdadc1* is on chromosome 14). The Harris test (10) can be used to assess the probability of the deviations from the expected Mendelian ratios. It relies on the χ^2 value, defined as the sum of the squares of deviations, divided by the expected numbers. Here, the χ^2 value is 8.4, and the number of degrees of freedom is 9-1=8. Therefore the probability for a deviation from the expected outcome as large or larger than observed is 1-cdf(χ^2,8.4,8)≈40%. Hence, the deviations from the expected numbers are not significant.

Supplementary Figures

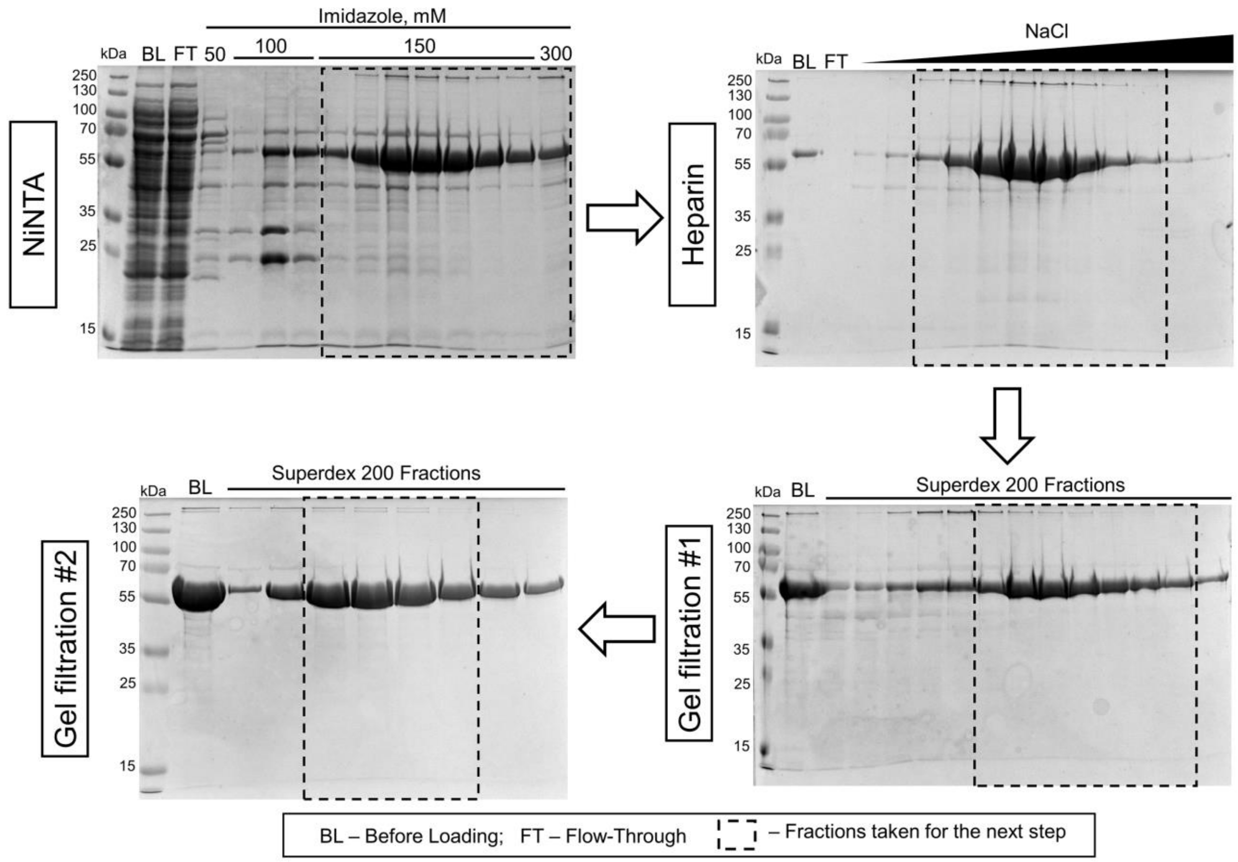

Fig. S1. Purification scheme of human CDADC1 and the SDS-PAGE analysis of CDADC1 fractions at each step.

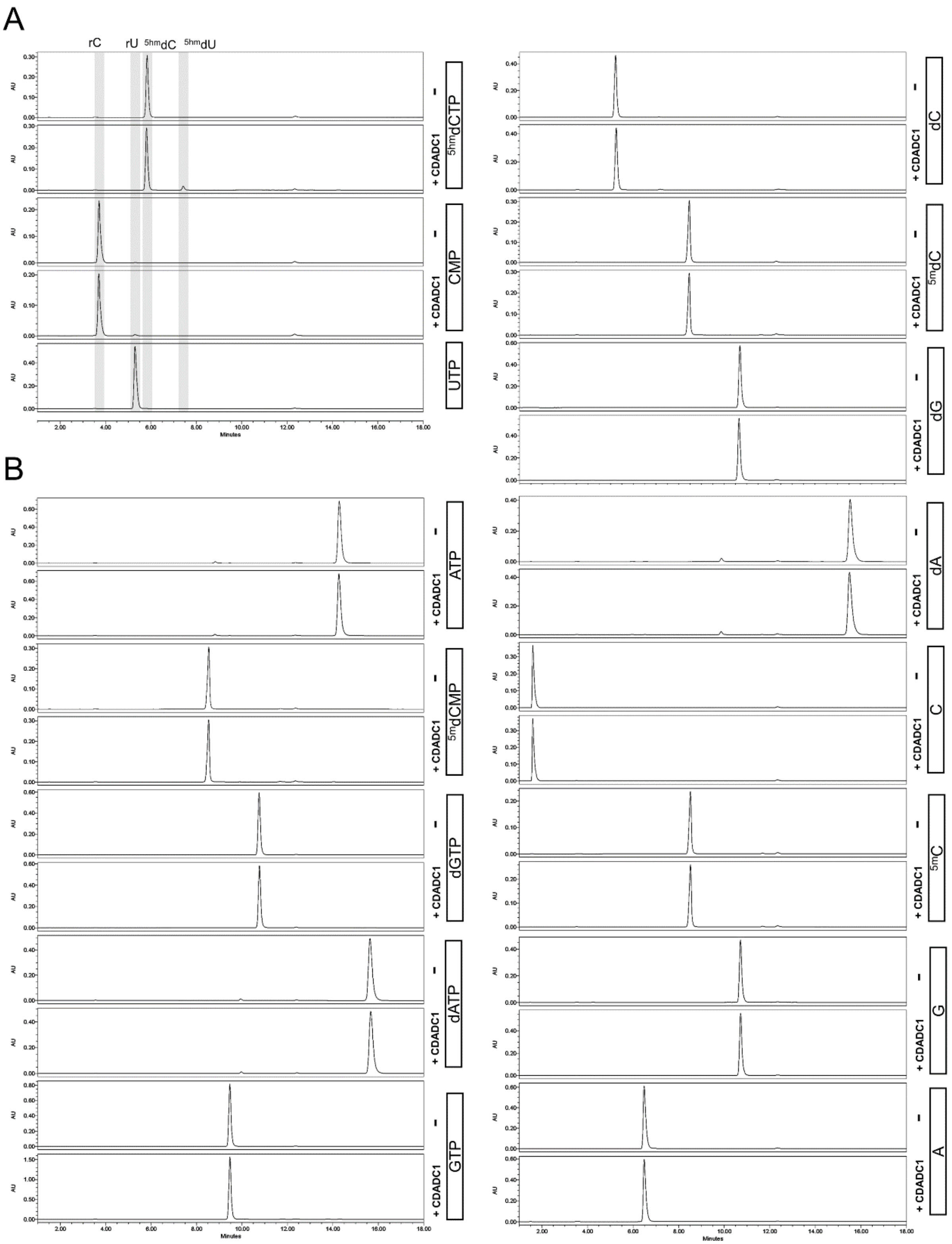

Fig. S2. UHPLC screen of CDADC1 activity. This figure is complementary to Fig 1. A) CDADC1 has a trace activity on ^{5hm}dCTP and CMP. B) All the compounds tested for which CDADC1 has no detectable activity. Note that all reaction substrates and products were dephosphorylated prior to UHPLC analysis.

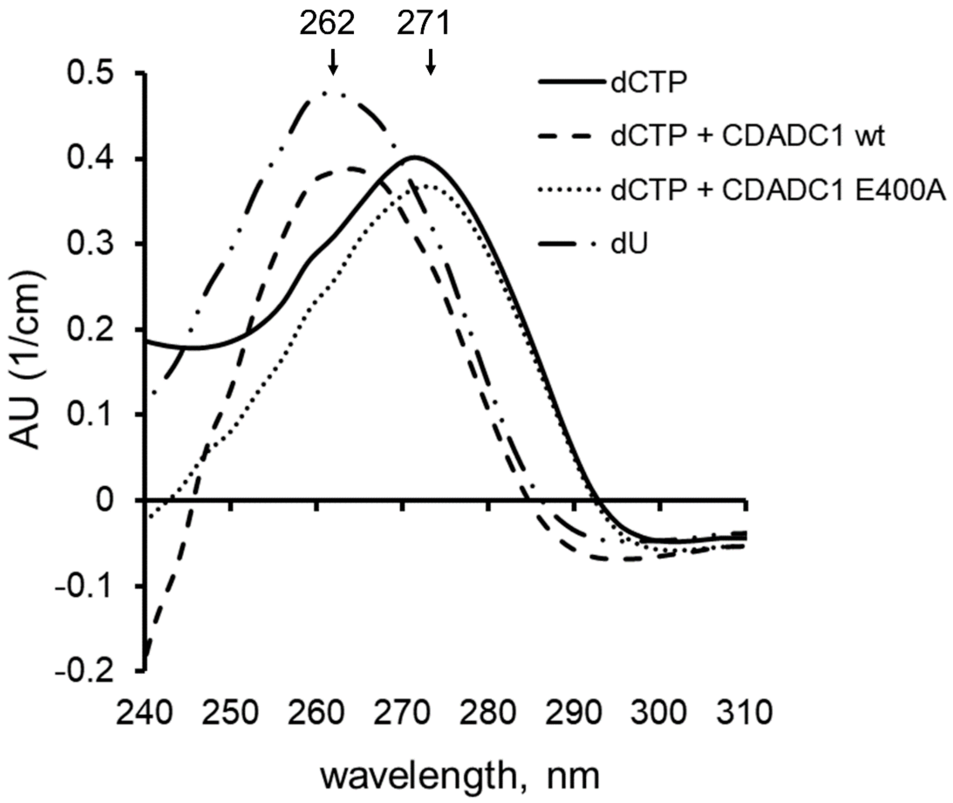

Fig. S3. CDADC1 E400A is catalytically inactive. Activity was measured using a spectrophotometric assay. The assay is based on a blue shift of the absorption maximum that accompanies the conversion of the amino pyrimidines (with electron donating amino substituent, absorption maximum ~271 nm) to keto-pyrimidines (with electron-withdrawing keto substituent, absorption maximum ~262 nm). dCTP was treated with the same amounts of CDADC1 wt or E400A, and the UV spectra of the reaction products were collected. dU absorbance was used as a reference.

```
CDADC1_NTD                    PRLSKVN--LFTLLSLWMELFPAE----AQRQKSQKNE----------------------    62
CDADC1_CTD                    ---------------EIARHCMVQARLLAYRTEDHKTGVGAVIWAEGKSRSCDGTGAMYF    361
DCTD_H_sapiens                --MSEVSCKKRDDYLEWPEYFMAVAFLSAQRSKDPNSQVGACIVNSE----------NKI    48
DCTD_S_mutans                 ----------MTNRLSWQDYFMANAELISKRSTCNRAYVGAVLVK-N----------NRI    39
DCTD_chlorovirus_PBCV_1       -------------MSKAEKFYSLACYHAQLFSKDPNTKVAALVIDNN----------NNI    37
DCTD_cyanophage_S_TIM5        ------------MKPEIKEAYMKTAELFSQVSNCKRMKVGAIVVK-N----------GSI    37
DCTD_phage_T4                 ---------------MKASTVLQIAYLVSQESKCCSWKVGAVIEK-N----------GRI    34
```

Allosteric G(Y/W)N(G/A) motif

```
CDADC1_NTD                    --EGK-HGPLGDNEERTRVSTDK----------------RQ---VK-RTGLVVVKNM-KI    98
CDADC1_CTD                    VGCGYNAFPVGSEYADFP--H-------------------------------MDDKQKD    387
DCTD_H_sapiens                VGIGYNGMPNGCSDDVLPWRRTA--------E--------------------------N    73
DCTD_S_mutans                 IATGYNGGVADTD-NCDDVGHEM-----------EDG----------------------    64
DCTD_chlorovirus_PBCV_1       ASVGYNGLPRGFEETSDRWEKP-------------------------------------    59
DCTD_cyanophage_S_TIM5        LAHGWNGTPSGFHTNCCE----L-----------EDG----------------------    59
DCTD_phage_T4                 ISTGYNGSPAGGV-NCCDYAAEQGWLLNKPKHAIIQGHKPECVSFGSTDRFVLAKEHRSA    93
                                 *
```

Catalytic HAE motif **PCxxC zinc coordination motif**

```
CDADC1_NTD                    VGLHCSSEDLHAGQIAL---IKHGSRLKNCDLYFSRKPCSACLKMIVNAGVNRISYWPAD    155
CDADC1_CTD                    REIRKFRYIIHAEQNALTFRCQEIKPEERSMIFVTKCPCDECVPLIKGAGIKQIYAGDVD    447
DCTD_H_sapiens                KLDTKYPYVCHAELNAIMN--KNSTDVKGCSMYVALFPCNECAKLIIQAGIKEVIFMSDK    131
DCTD_S_mutans                 ---H-CIRTVHAEMNALIQCAKEGISANNTEIYVTHFPCINCTKALLQAGVKKITYNTAY    120
DCTD_chlorovirus_PBCV_1       ---MKYNYVVHAEANAIATAARNGVRLDGCSIITTLFPCKECSKLIIQSGIRKVITSKPC    116
DCTD_cyanophage_S_TIM5        ---STNPFVLHAEQNALVKMAKSSESIDGSELFCTHSPCPDCSKMIAQAGVKKVYYRNEY    116
DCTD_phage_T4                 HSEWSSKNEIHAELNAILFAARNGSSIEGATMYVTLSPCPDCAKAIAQSGIKKLVYCETY    153
                                        **   *:    :     .   :  :  **  *   :  :*:..:
```

```
CDADC1_NTD                    PEISLLTEASSSEDAKLDAKAVERLK------------SNSRAHVC-----    189
CDADC1_CTD                    VGK-----KKADIS-YMRFGELE---------GVSKFTWQLNPS-------    476
DCTD_H_sapiens                YHDSD--EATAARL-LFNMAGVTFRKF-IPKCSKIVIDFDSINSRPSQKLQ    178
DCTD_S_mutans                 RIH------PFAIE-LMTQKEVEYVQHDVPRVKLGEK--------------    150
DCTD_chlorovirus_PBCV_1       KDSSWLESFSFSNE-MFDECGIEVEYL------------------------    142
DCTD_cyanophage_S_TIM5        RIT------DG-ID-VLQQLGVEVEKM------------------------    135
DCTD_phage_T4                 DKN-----KPGWDD-ILRNAGIEVF--NVPKKNLNKLNWENINEFCGE---    193
                                              :    :
```

Fig. S4. Amino acid sequence alignment of human CDADC1 NTD and CTD with diverse DCTDs. Key motifs are highlighted. The mutations in both catalytic and allosteric sites in the CDADC1 NTD are indicated in red. DCTDs were selected for comparison based on the availability of the biochemical and structural data that confirm functional relevance of the conserved motifs: *H. sapiens* (PDB: 2W4L, (11)), *Streptococcus mutans* (PDB: 2HVW, (12)), *Paramecium bursaria* Chlorella virus 1 (PDB: 7FH4), (13)), cyanophage S-TIM5 (PDB: 4P9C, (14)), phage T4 (PDB: 1VQ2, (15)). Sequences were aligned using Clustal Omega (16).

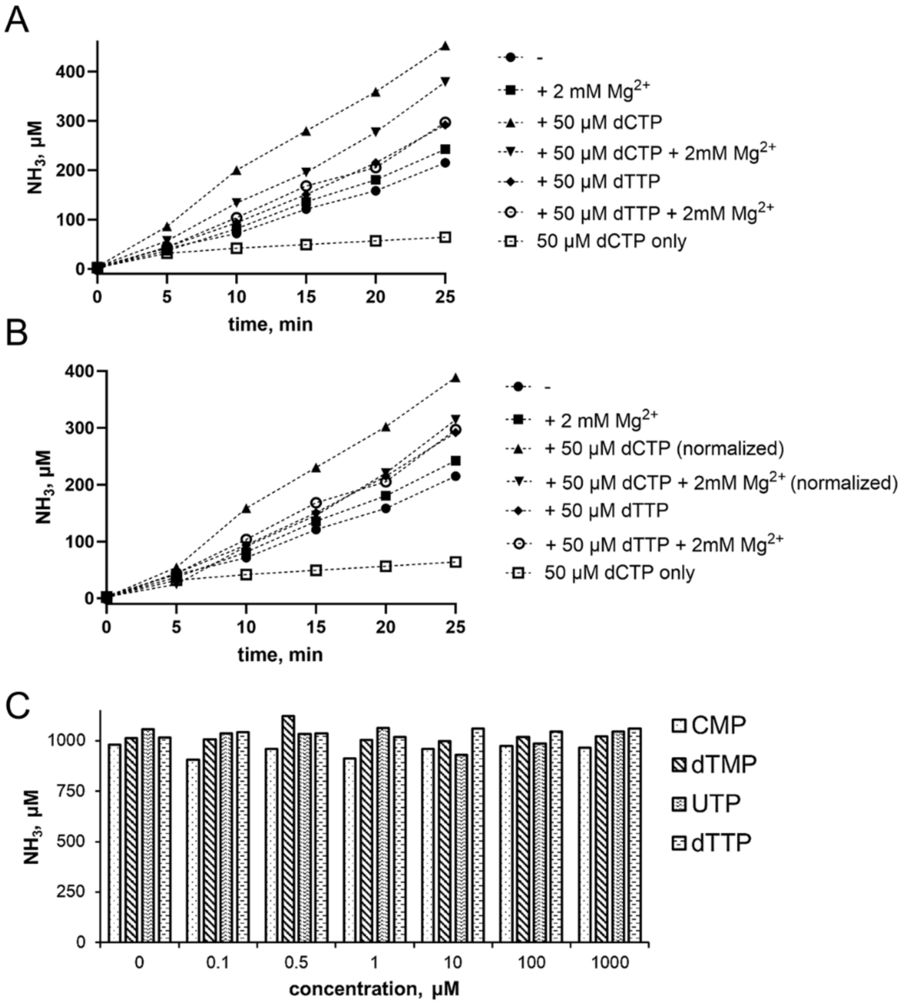

Fig. S5. Test of the allosteric regulation of CDADC1 activity. A) Kinetics of CDADC1 catalyzed deamination of dCMP (1 mM) in the presence or absence of dCTP/dTTP (50 μM) and Mg^{2+} (2 mM). B) The same graph as in panel A, but the ammonia released in dCTP deamination is subtracted from the ammonia released from dCMP. C) CDADC1 activity on dCTP in the presence of different concentrations of CMP, dTMP, UTP, and dTTP.

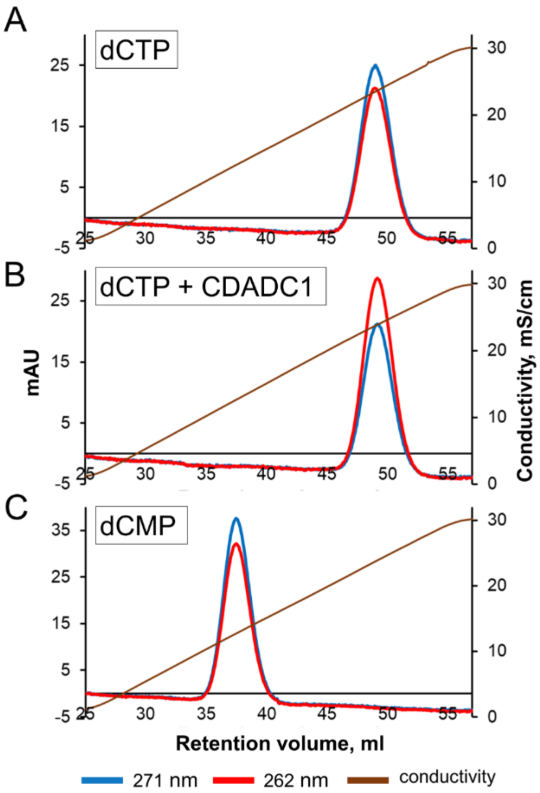

Fig. S6. Test for putative phosphatase activity of CDADC1. dCTP incubated in the absence (A) or in the presence (B) of CDADC1 was applied on Q-sepharose and eluted with a linear NaCl gradient. dCMP was used as a reference of the weaker binding to the resin due to the presence of only one phosphate group (C). Elution at similar ionic strength of samples treated and untreated with CDADC1 and the absorbance measured at A_{max} of both cytosine (271 nm) and uracil (262 nm) strongly suggests that CDADC1 deaminates dCTP, but does not dephosphorylate it.

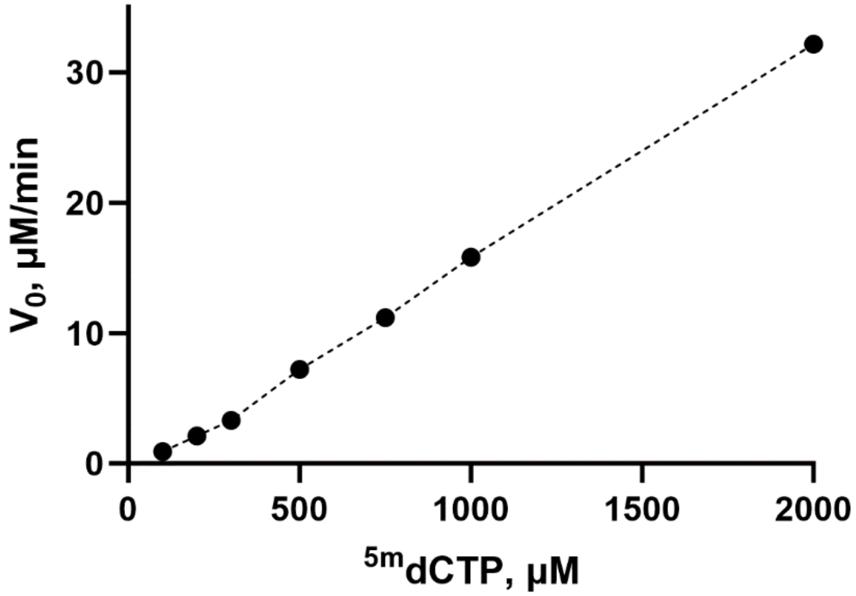

Fig. S7. Measurement of CDADC1 activity at different concentrations of ^{5m}dCTP. V_0 is plotted against ^{5m}dCTP concentration. The graph shows that at 2 mM ^{5m}dCTP CDADC1 is still far from achieving its V_{max} with this substrate.

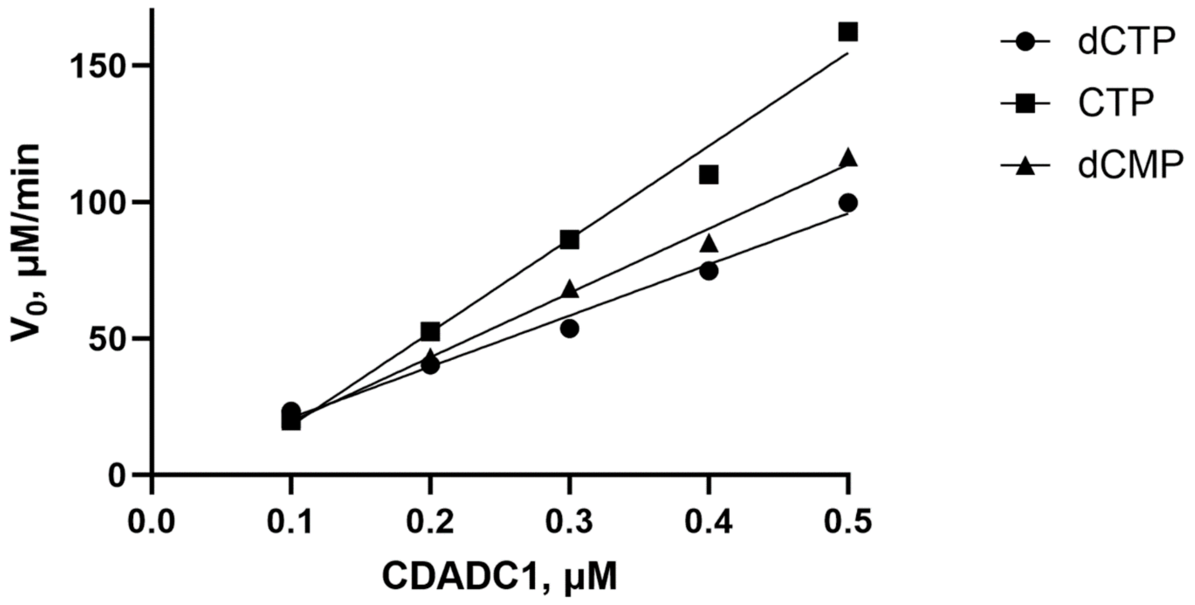

Fig. S8. Test of the dynamic CDADC1 concentration range in the presence of saturating substrate. The rate of ammonia release was measured in the presence of 5 mM dCTP, CTP, or dCMP. Initial velocities are plotted against CDADC1 concentration.

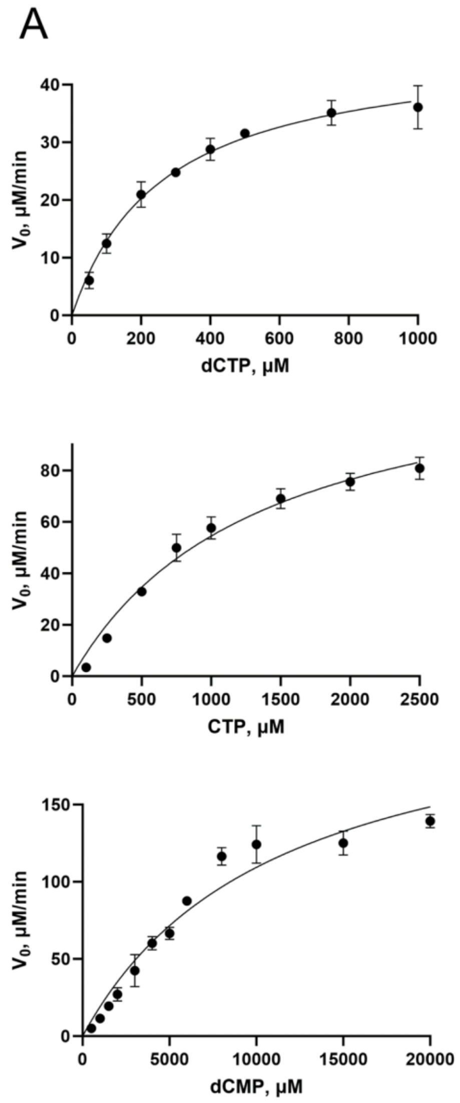

B

	dCTP	CTP	dCMP
**Allosteric sigmoidal**			
Best-fit values			
V_{max}	41.76	89.54	152.4
h	1.222	1.618	1.748
K_{half}	206.0	685.2	5042
K_{prime}	671.2	38679	2966648
95% CI			
V_{max}	37.56 - 49.11	83.18 - 98.77	141.6 - 166.8
h	0.9631 - 1.508	1.370 - 1.901	1.497 - 2.033
K_{half}	164.5 - 295.2	606.9 - 811.0	4512 - 5827
Goodness of Fit			
Degrees of Freedom	21	25	41
R squared	0.9721	0.9881	0.9760
Sum of Squares	69.85	241.3	2183
Sy.x	1.824	3.107	7.297
Constraints			
h	h > 0	h > 0	h > 0
K_{half}	$K_{half} > 0$	$K_{half} > 0$	$K_{half} > 0$
**Michaelis-Menten**			
Best-fit values			
V_{max}	47.00	128.2	231.6
K_M	264.0	1354	11190
95% CI			
V_{max}	43.46 to 51.15	114.3 to 146.3	202.9 to 268.7
K_M	212.6 to 328.2	1062 to 1752	8756 to 14505
Goodness of Fit			
Degrees of Freedom	22	26	42
R squared	0.9680	0.9730	0.9493
Sum of Squares	80.30	549.2	4611
Sy.x	1.910	4.596	10.48
Constraints			
K_M	Km > 0	Km > 0	Km > 0
Number of points			
# of X values	40	80	140
# Y values analyzed	24	28	44

Fig. S9. Comparison of Michaelis-Menten and cooperative models for analysis of CDADC1 kinetic data. This figure is complementary to Fig. 1. A) V_0 plotted against corresponding substrate concentration fitted in the M-M model. B) Interpretation in terms of an allosteric sigmoidal (top) and standard Michaelis-Menten (bottom) model.

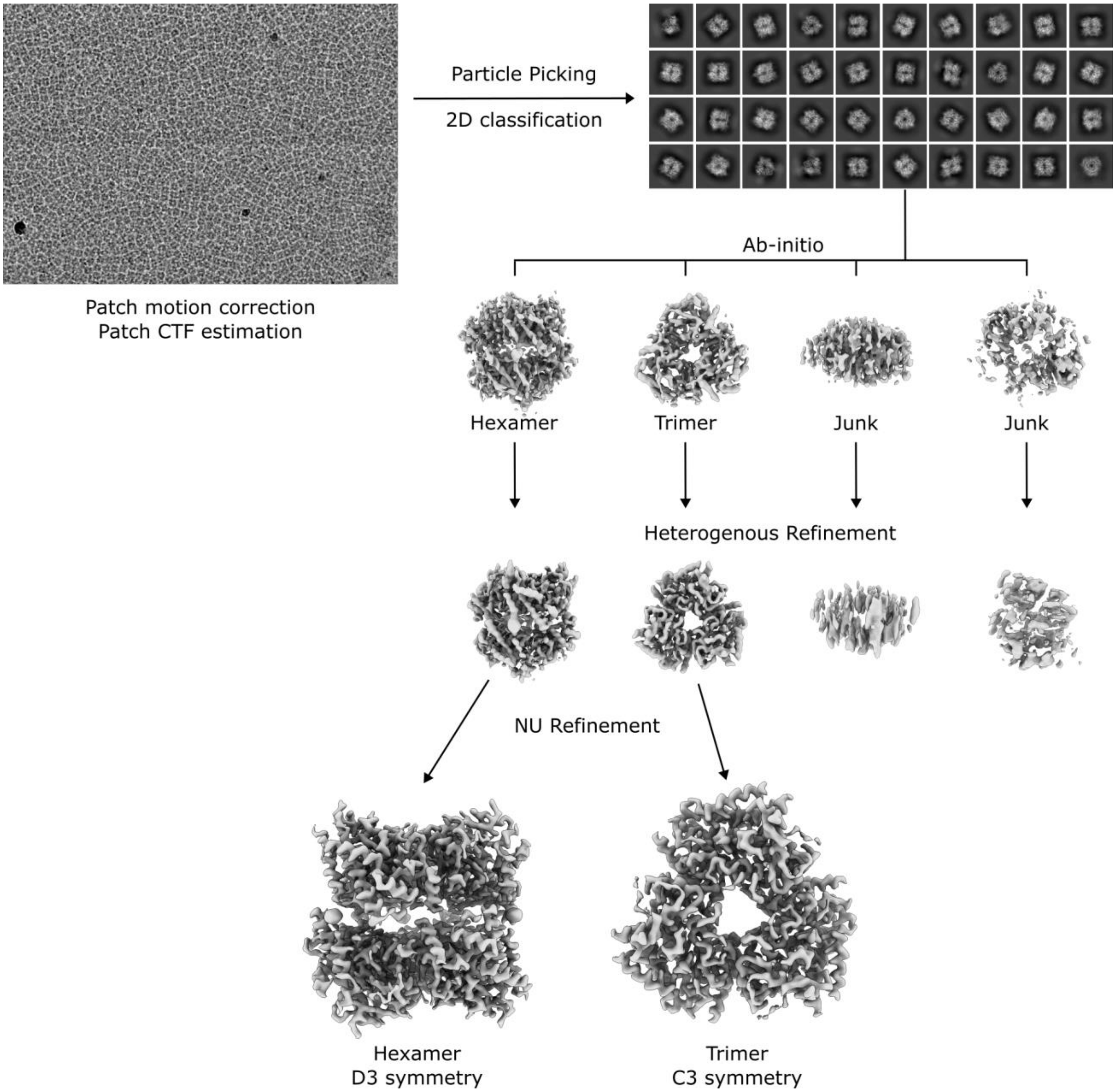

Fig. S10. Cryo-EM data processing workflow. “NU Refinement” stands for non-uniform refinement.

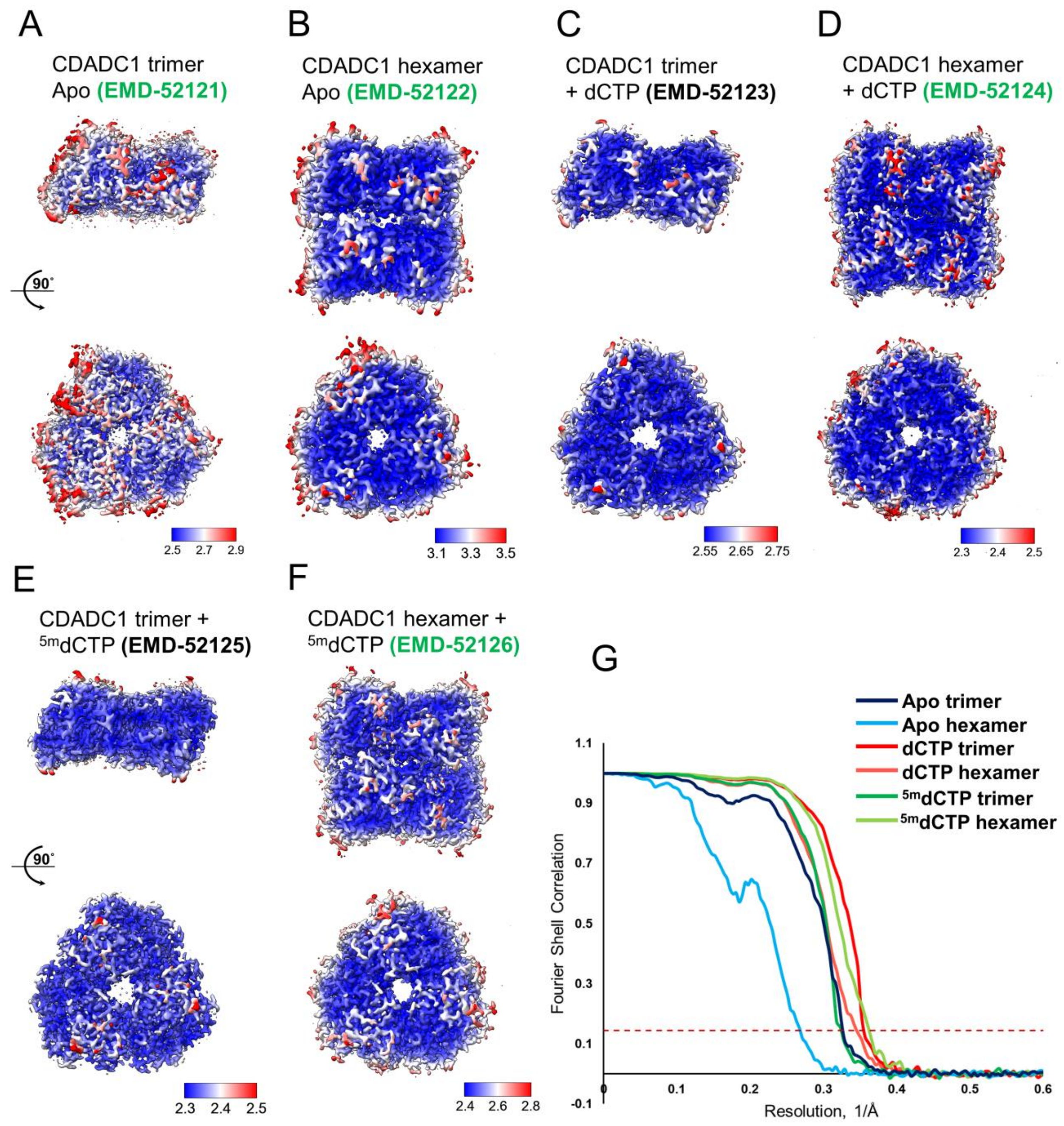

Fig. S11. Visualization of map quality. A-F) ESP maps colored according to local resolution. Maps were submitted to the EMDB for both trimers and hexamers. For the map accessions indicated in green, models were also submitted to the PDB. G) Fourier Shell Correlation (FSC) as a function of inverse resolution for the trimer and hexamer maps.

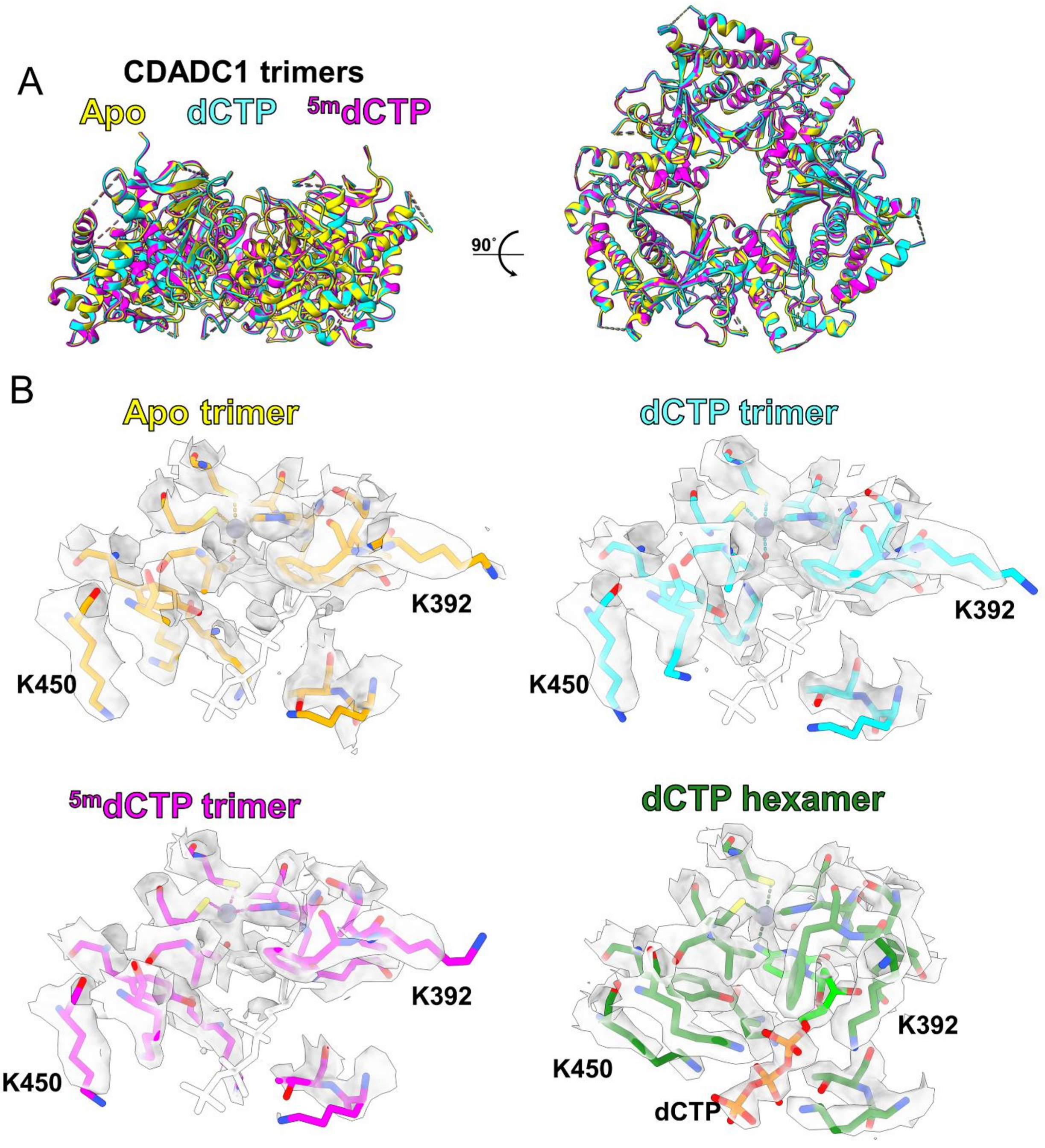

Fig. S12. Comparison of CDADC1 E400A trimer structures. A) Alignment of the trimer structures showing that they are nearly identical, irrespective of whether dCTP or 5mdCTP was present in the buffer or not. B) Comparison of the active sites of the trimers. Protein residues are colored as in A. The active site of the hexamer (green) with bound dCTP (lime) is shown as a reference. ESP maps are shown as transparent gray surfaces. The “mock” dCTP (white) in the active sites of trimers is shown to highlight the absence of ligand densities.

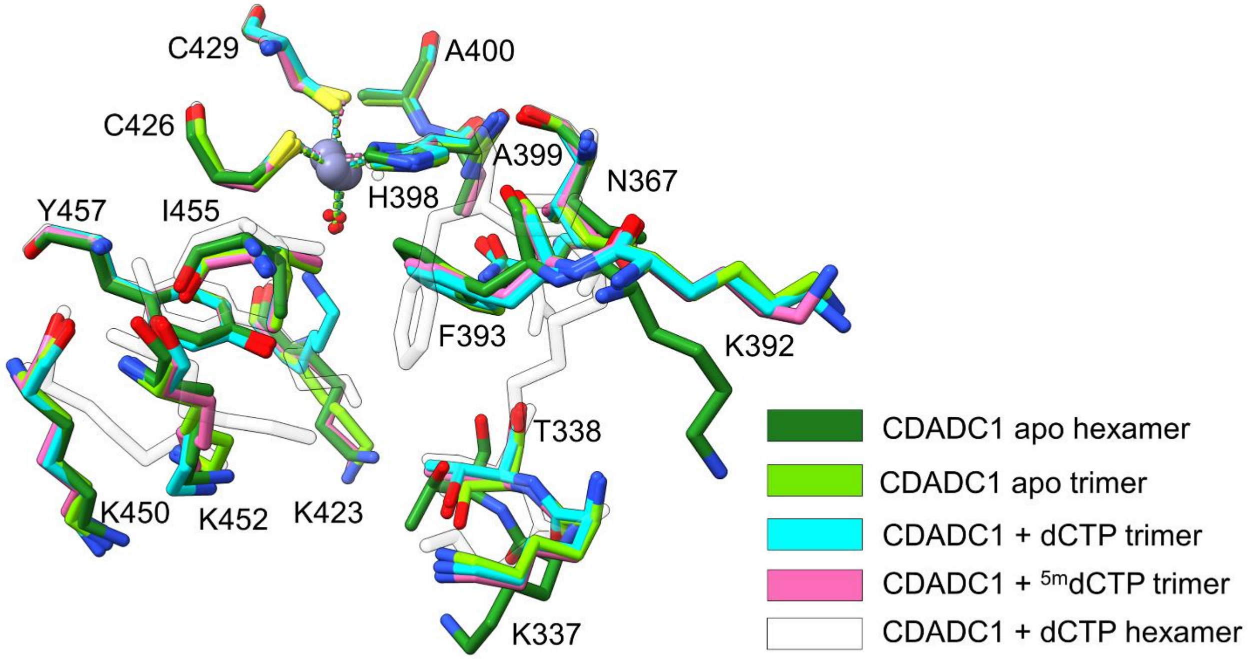

Fig. S13. Superposition of the active sites of CDADC1 trimer and hexamer in the absence of substrate with active sites of CDADC1 trimers reconstructed from the datasets with dCTP and ^{5m}dCTP. As reference, the outlines of active site residues of CDADC1 hexamer with bound dCTP (dCTP not shown) are provided. The figure shows no significant difference between the compared active sites, confirming the absence of ligands in CDADC1 trimers.

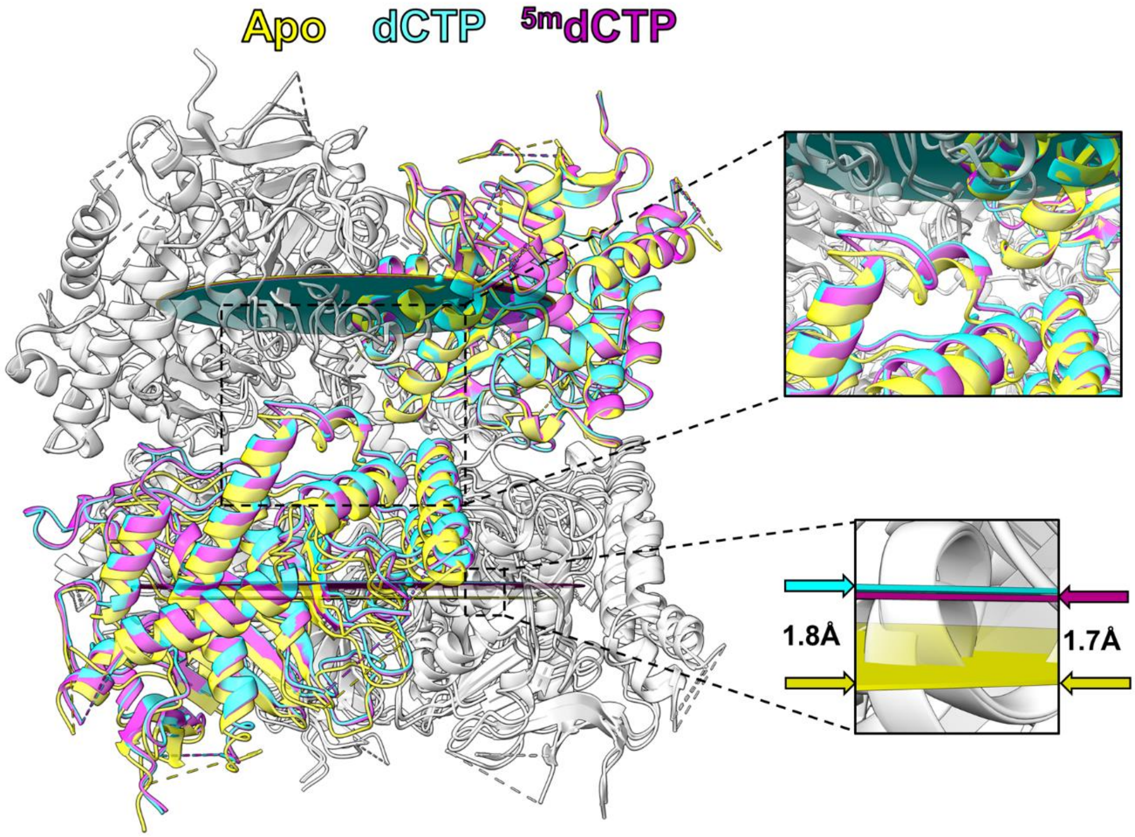

Fig S14. Comparison of CDADC1 hexamers without bound ligand, and in the complexes with dCTP and ^{5m}dCTP. The planes are drawn between zinc atoms in the NTDs of the protomers that make up each of the two trimers in the hexamer. Upon ligand binding, the trimers in the hexamer come closer together. The effect is more pronounced for the stronger ligand dCTP, and slightly less pronounced for the weaker ligand ^{5m}dCTP.

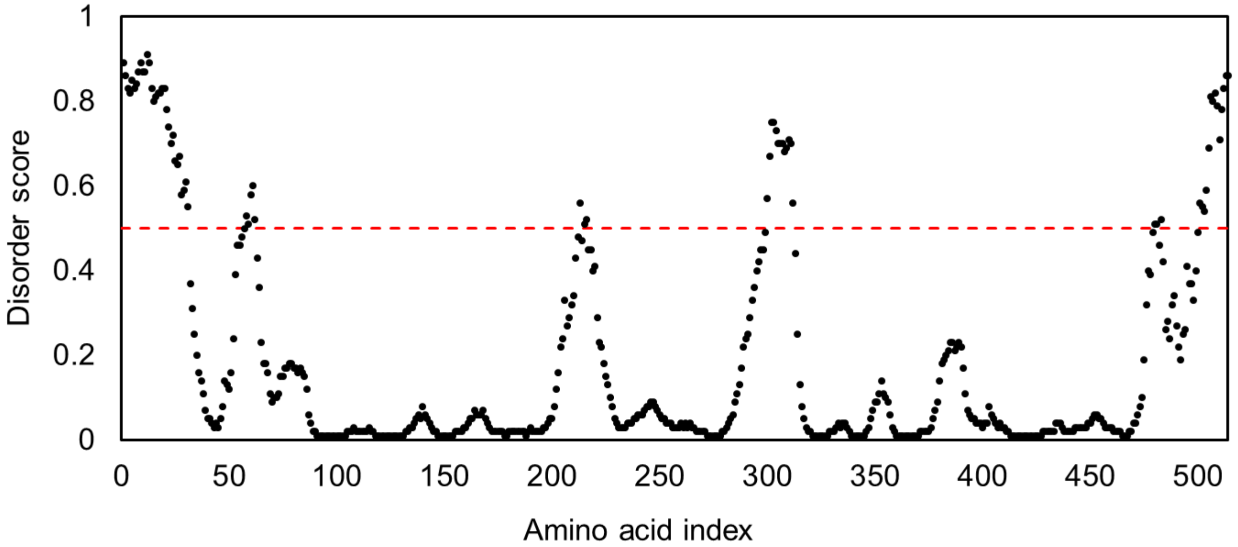

Fig. S15. Prediction of the intrinsically disordered regions in human CDADC1 using DISOPRED 3 server (17).

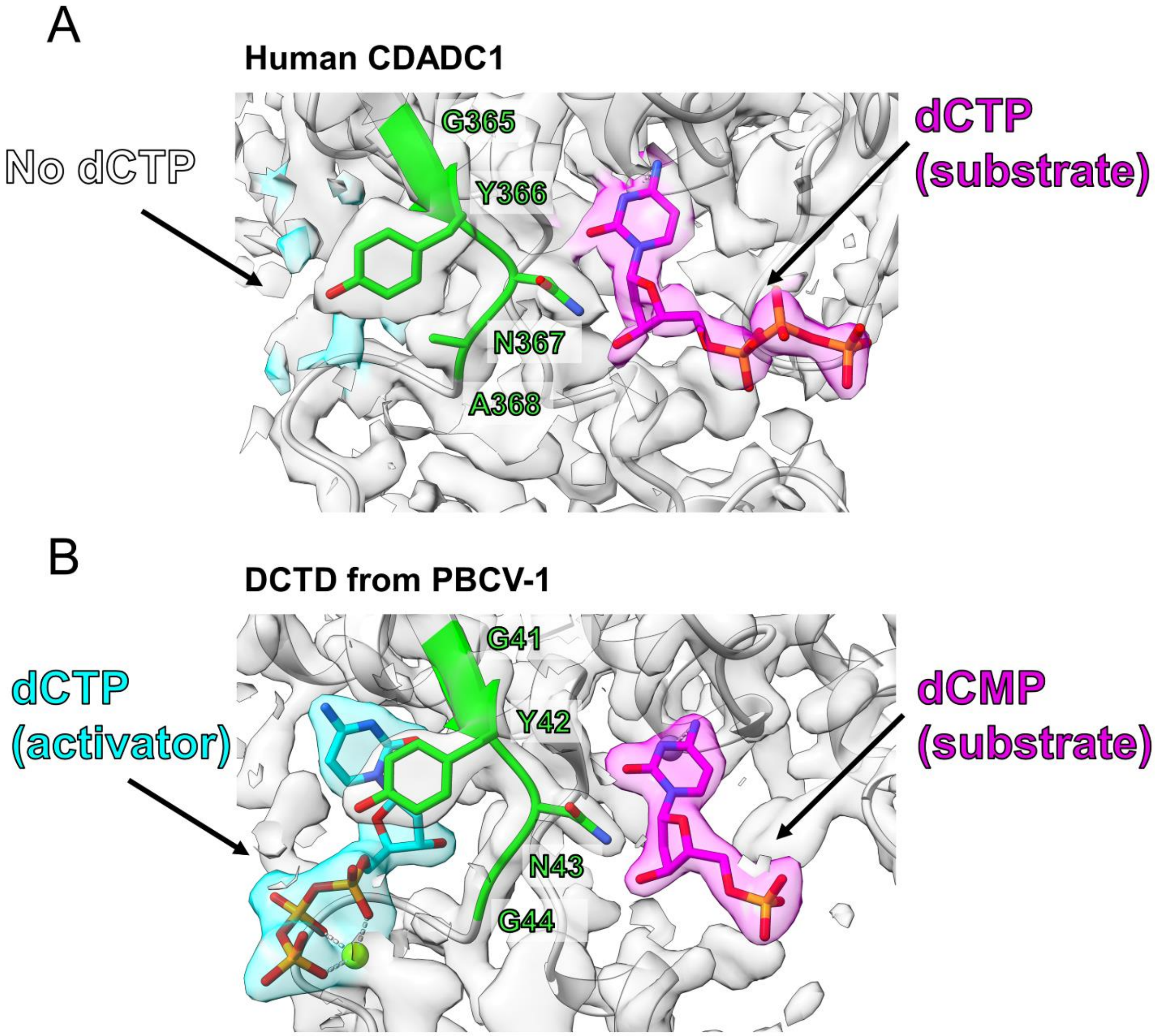

Fig. S16. Putative allosteric site in CDADC1. A) CDADC1 E400A contains dCTP in the active site. B) DCTD from PBCV-1 contains dCMP (substrate) in the active site and dCTP (activator) in the allosteric site. The proteins are shown in analogous orientations and the conserved allosteric site residues are colored green. To highlight the absence of dCTP in the putative allosteric site of CDADC1, the DCTD from PBCV-1 was superposed on CDADC1 CTD and the ESP map around the DCTD bound dCTP was colored cyan.

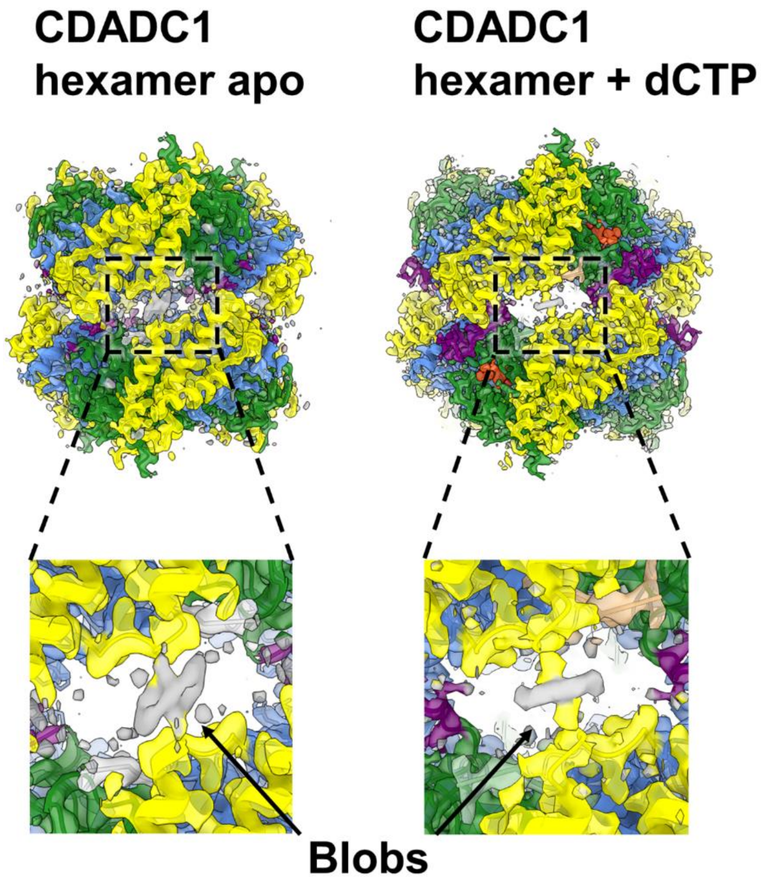

Fig. S17. Blobs of density at the interface of two trimers in a hexamer that are not obviously attributable to any part of CDADC1.

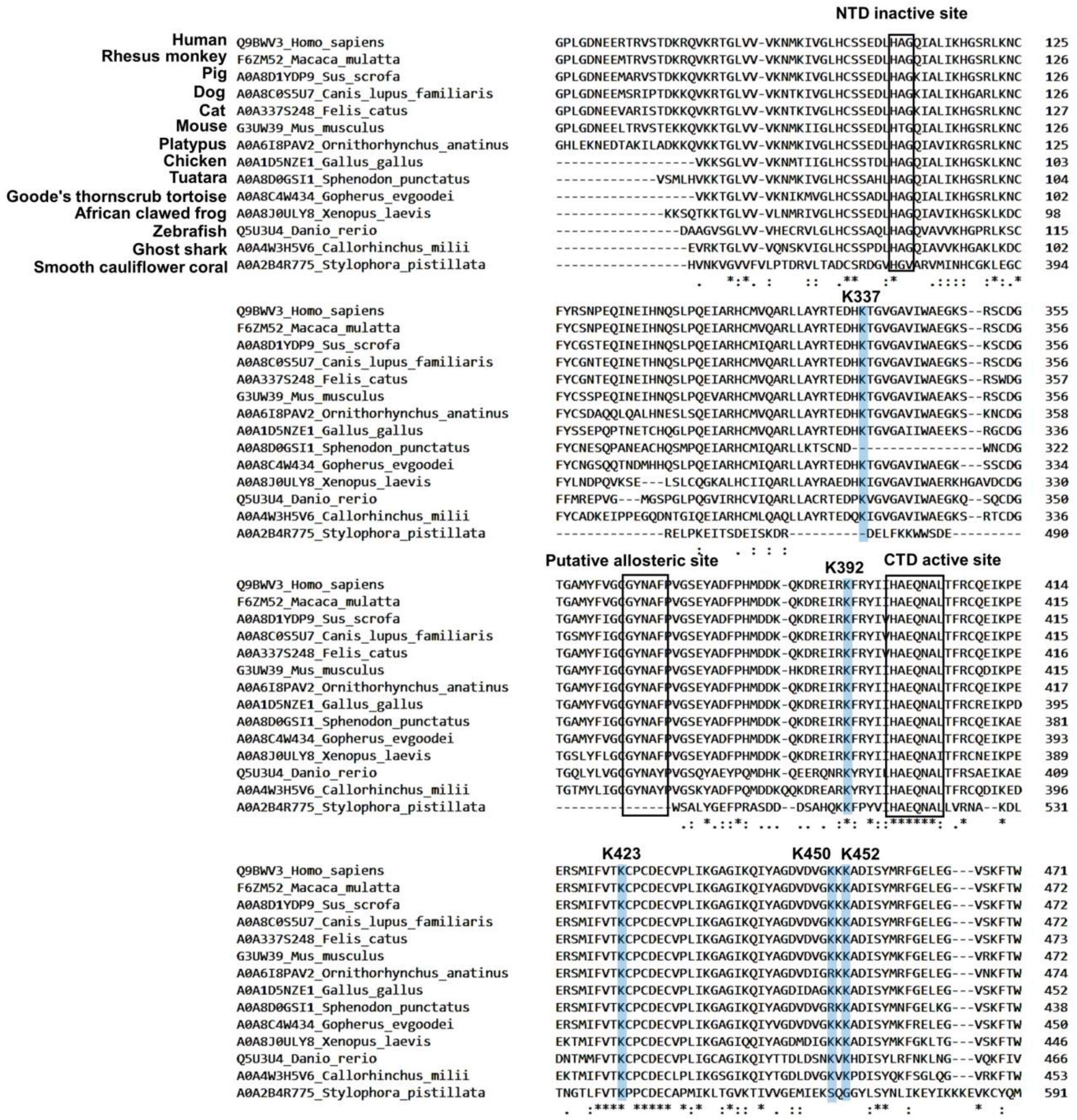

Fig. S18. Amino acid sequence conservation of CDADC1 in different species. The relevant regions are highlighted by black boxes. Lysine residues involved in binding of the triphosphate tail of dCTP are highlighted in blue. Numeration is according to the human CDADC1 sequence. Sequences were aligned using Clustal Omega (16). CDADC1 is present in jawed vertebrates, including cartilaginous fishes, the oldest fossils of which are ~ 439 million year old (18). Surprisingly, it is absent in jawless fishes and any other early vertebrates, such as lancelets. However, CDADC1-like proteins can be found in many invertebrates, such as cnidarians (e.g. *S. pistillata*). Therefore, it is possible that vertebrate and invertebrate CDADC1 proteins have separate origins.

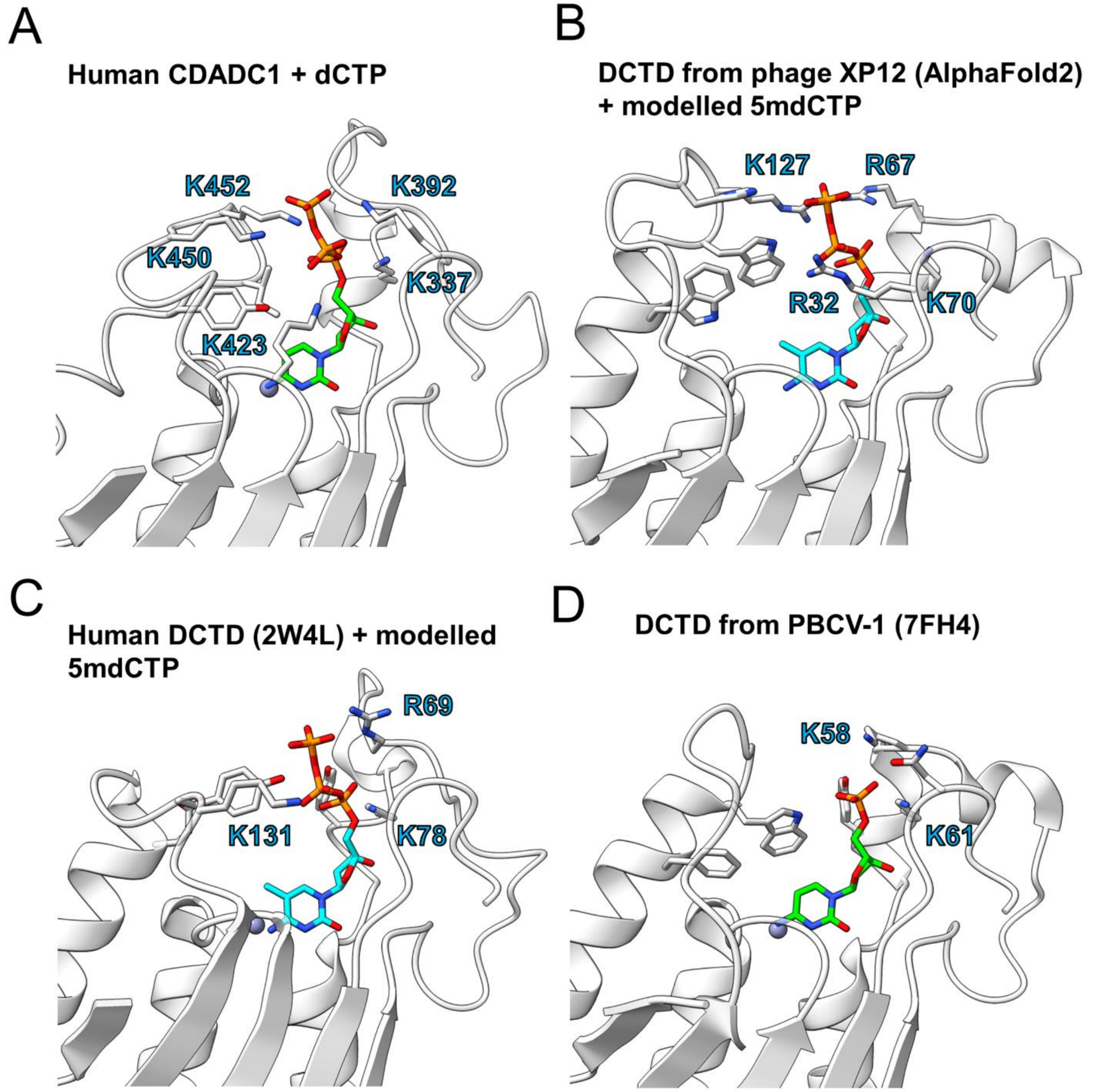

Fig. S19. Positively charged lysine and arginine residues at the “entry” to the ligand binding pocket. A) Human CDADC1. B) DCTD from XP-12. C) Human DCTD and D) DCTD from PBCV-1. The comparison shows that CDADC1 has the greatest accumulation of positive charge in the region that would be expected to interact with the triphosphate tail of dCTP or ^{5m}dCTP. The DCTD proteins from XP-12 and PBCV-1 can deaminate triphosphates.

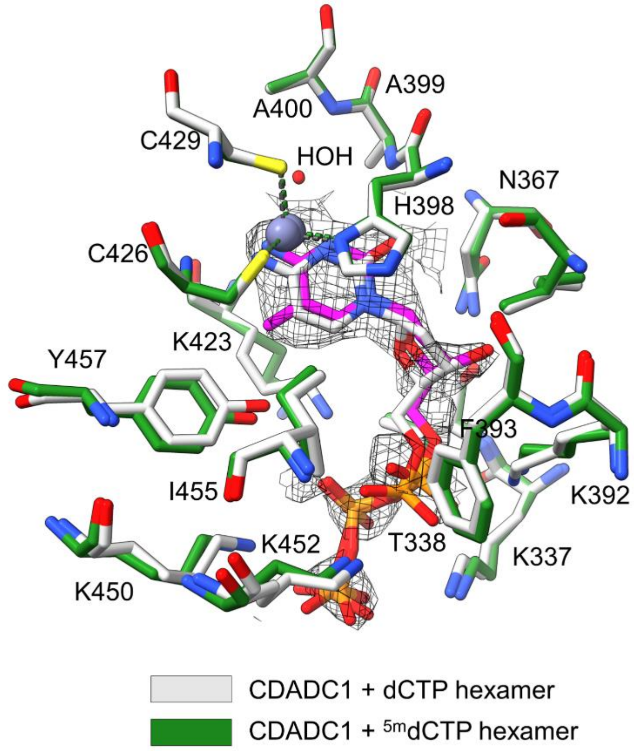

Fig. S20. Superposition of the CDADC1 active sites with bound dCTP and ^{5m}dCTP. The EP density that corresponds to ^{5m}dCTP is shown as gray mesh.

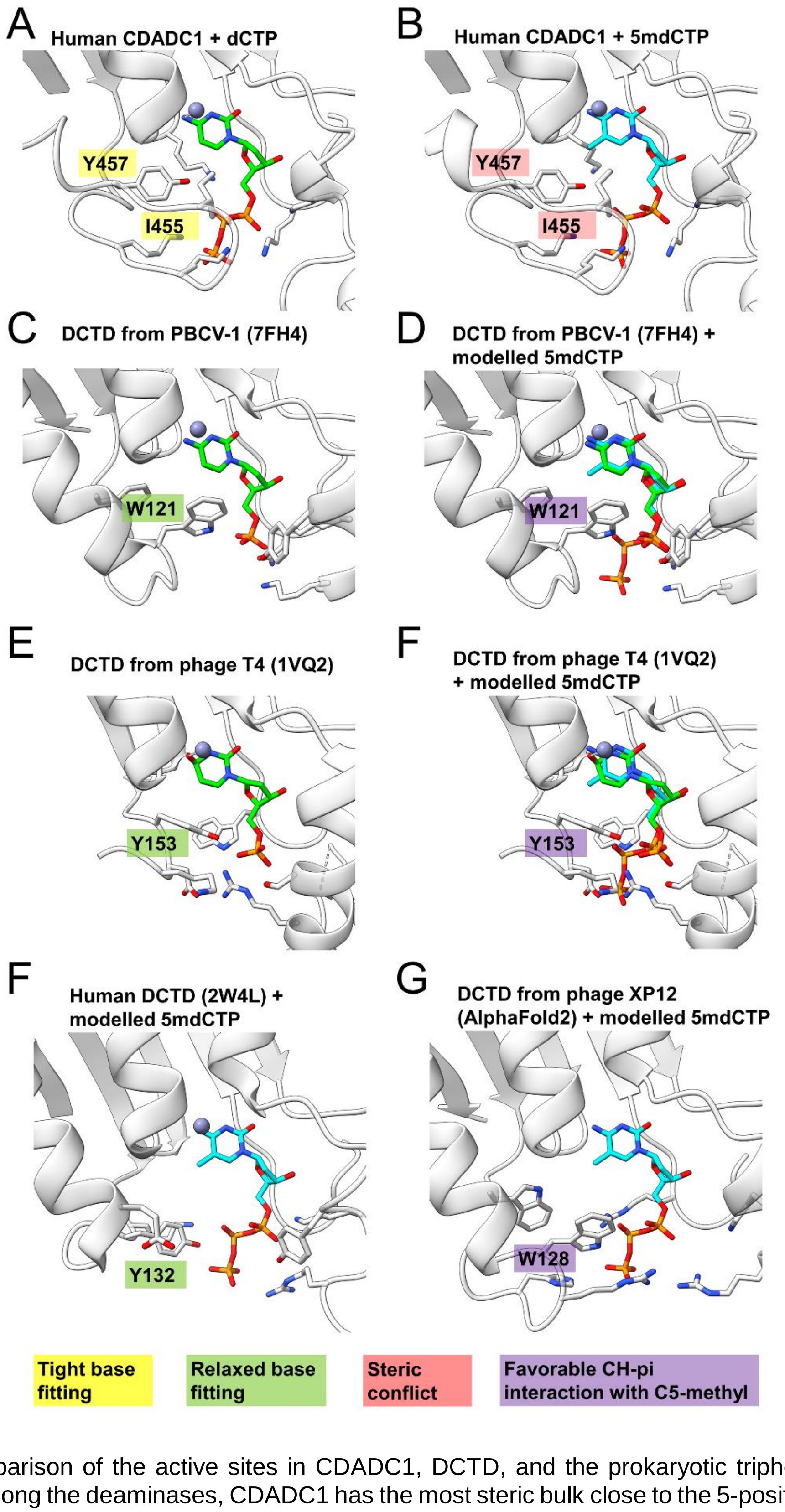

Fig. S21. Comparison of the active sites in CDADC1, DCTD, and the prokaryotic triphosphate-specific deaminases. Among the deaminases, CDADC1 has the most steric bulk close to the 5-position of the bound pyrimidine ligand, suggesting that CDADC1 has a stronger preference for dCTP over ^{5m}dCTP than DCTD proteins.

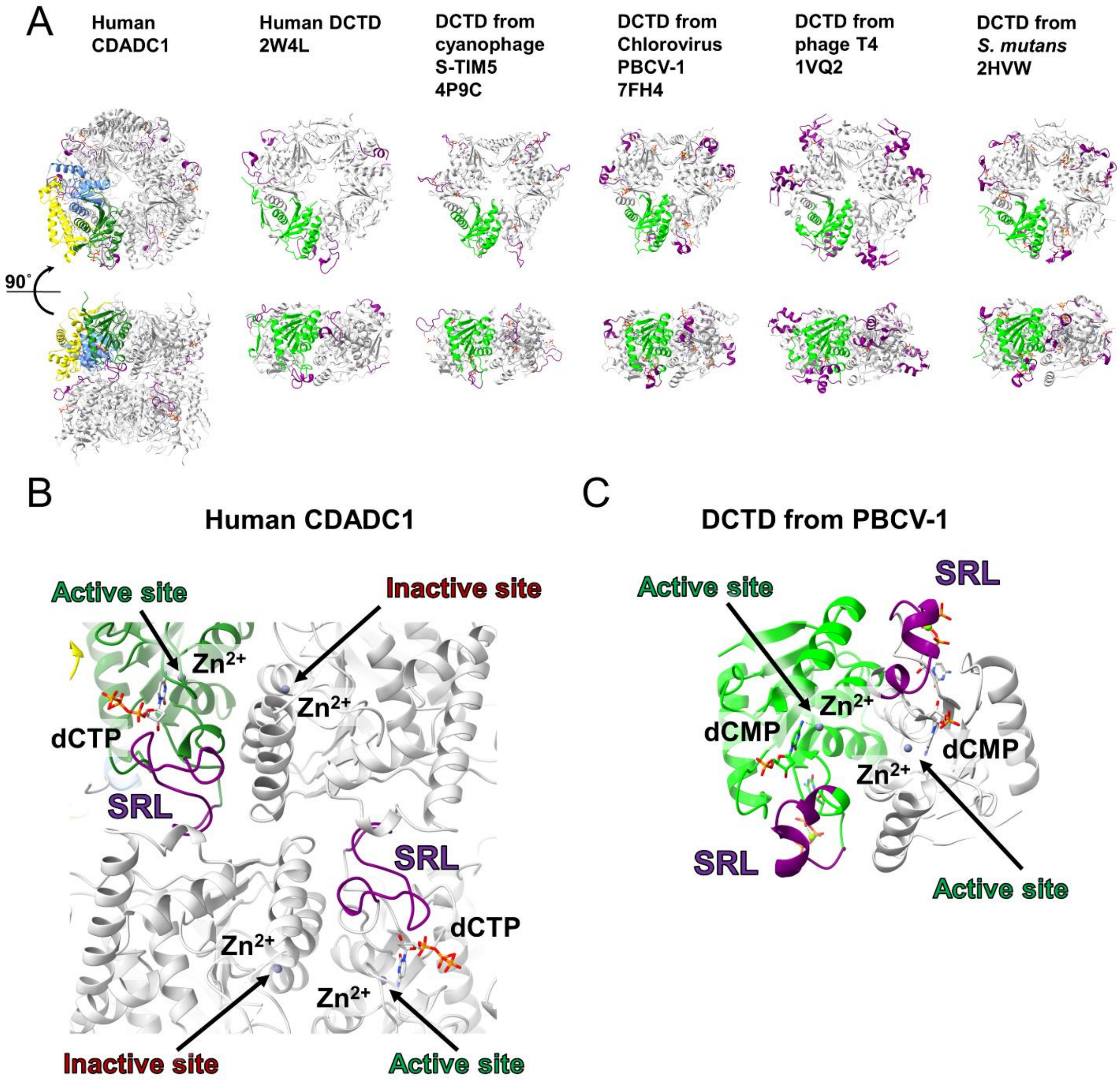

Fig S22. Comparison of the overall structure of CDADC1 with the structures of DCTD proteins from various sources. A) Overall structures. In CDADC1, the single protomer is colored according to the domain: NTD - blue, linker - yellow, CTD - green. In other DCTDs, the single protomer is colored light green. SRL in CDADC1 (res: 373-391) and SRL-like structures in other DCTDs (2w4l res: 60-77; 4p9c res: 49-62; 7fh4 res: 49-62; 1vq2 res: 45-96; 2hvw res: 50-66) are colored purple in every protomer. B) Relative localization of the active and inactive zinc-coordinating sites and SRLs in CDADC1 hexamer with bound dCTP in the active sites. C) Relative localization of the active sites and SRL-like regions in PBCV-1 DCTD hexamer in with bound dCMP in the active sites, and dCTP in the allosteric sites.

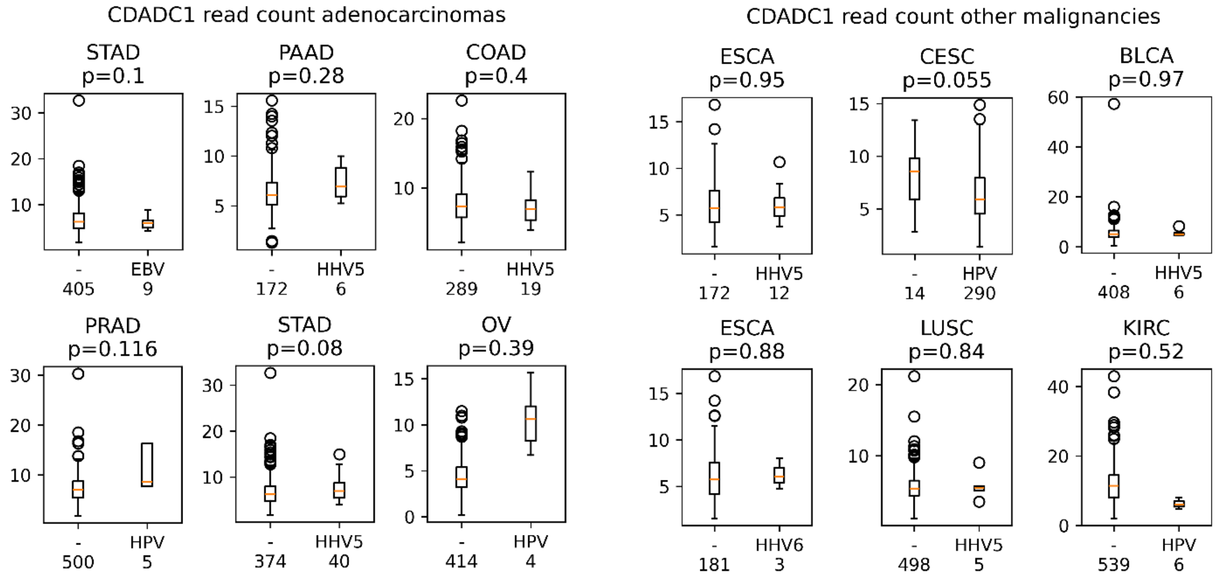

Fig S23. CDADC1 transcript count (in TPM, thousands per million) in virus infected and non-infected ("-") human adenocarcinoma cell lines (left) and non-adenocarcinoma cell lines (right). Malignancy abbreviations: STAD Stomach adenocarcinoma; PAAD Pancreatic adenocarcinoma; COAD Colon adenocarcinoma; PRAD Prostate adenocarcinoma; OV Ovarian serous cystadenocarcinoma; ESCA Esophageal carcinoma; CESC Cervical squamous cell carcinoma; BLCA Bladder Urothelial Carcinoma; LUSC Lung squamous cell carcinoma; KIRC Kidney renal clear cell carcinoma. Virus abbreviations: EBV Epstein Barr virus; HBV Hepatitis B virus, HHV5 Cytomegalovirus, HPV Human Papillomavirus; HCV Hepatitis C virus; HHV6 Human Herpes virus 6. The p-values are for a two-sided t-test (not assuming equal variance), before applying the Bonferroni correction for multiple hypothesis testing. Numbers below virus types and uninfected controls ("-") indicate the number of samples in the analysis.

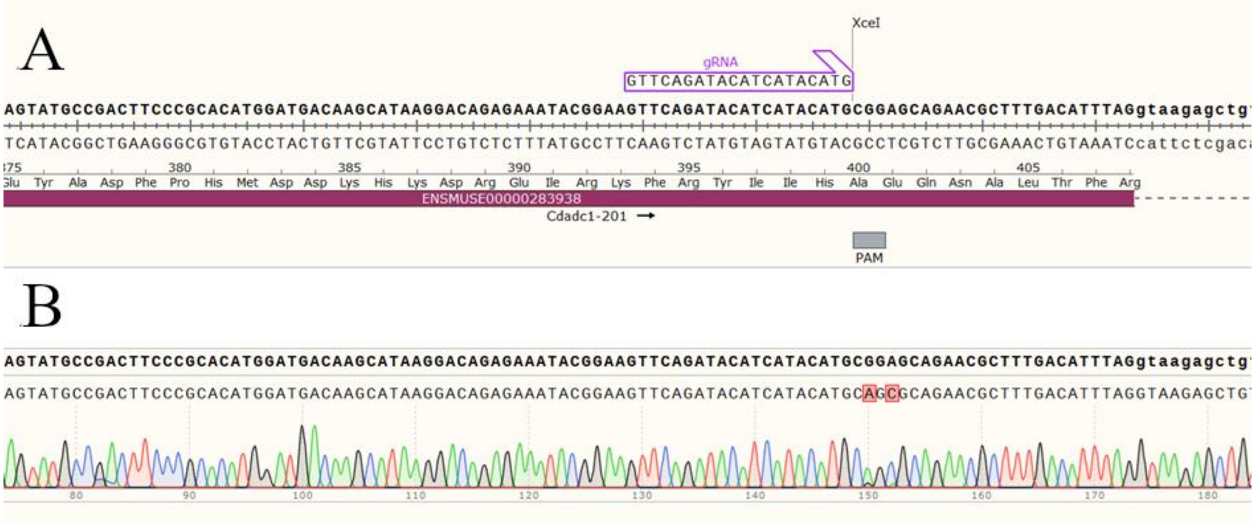

Fig. S24. Introduction of the *Cdadc1* E401A mutation in mouse (equivalent to the E400A mutation in human CDADC1). A) Design of the guide RNA (gRNA). The purple arrow shows the position of the Cas9 gRNA. The sense strand is in bold, the antisense strand in normal font. B) Validation of the intended sequence change. The WT sequence is in bold, the experimentally observed sequence is in normal font. The right red box highlights the A→C mutation that changes a GAG encoding glutamate to GCG encoding alanine. The left red box highlights the G→A mutation that changes a GCG to a GCA codon. This change is silent as both codons code for alanine. The change was introduced to destroy the NGG PAM, so that the repaired DNA is not susceptible to Cas9 cleavage.

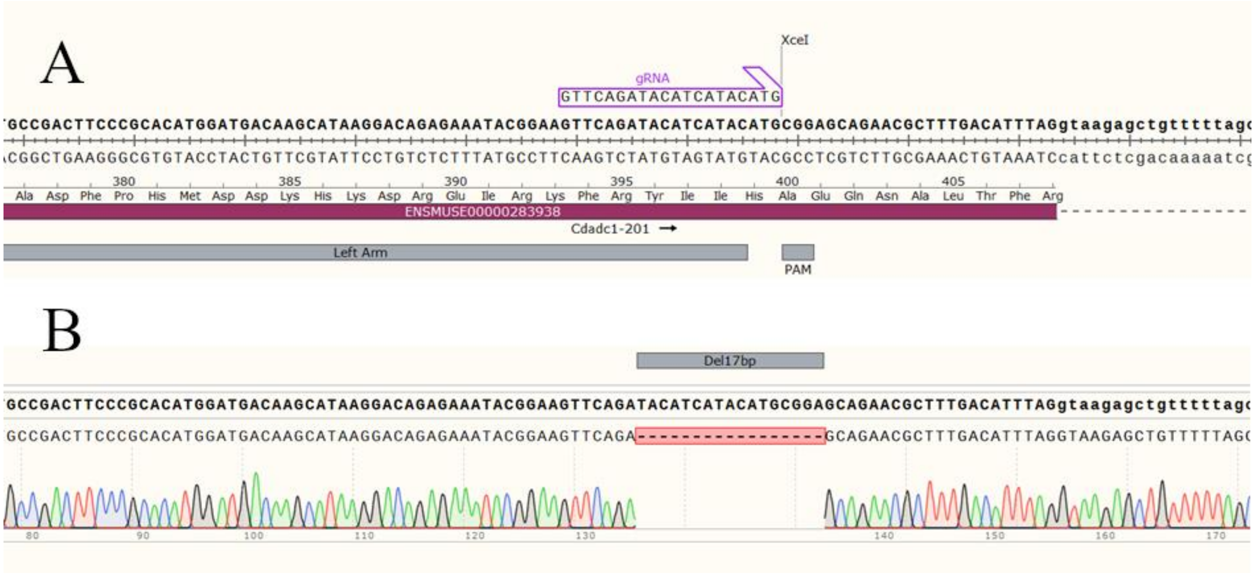

Fig. S25. Introduction of the deletion in *Cdadc1*. A) The same CRISPR guide RNA (gRNA) as for the introduction of the E401A mutation was used, but without the repair template. B) Imprecise repair generated a 17 bp deletion. The sequence in bold is the WT sequence, the sequence in normal font is the experimentally observed sequence.

A

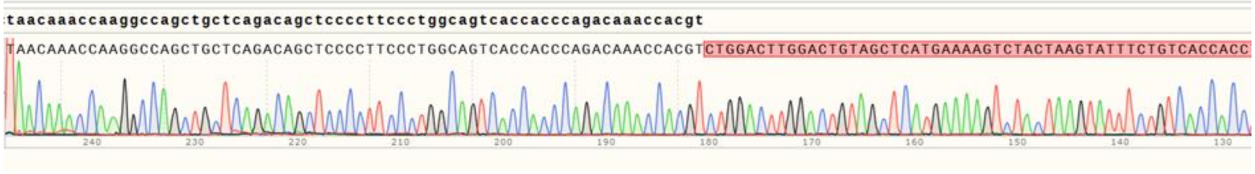

B

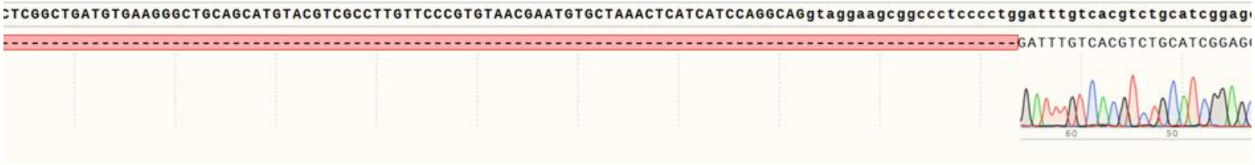

Fig. S26. Sequencing result for the deletion of the third coding (overall fourth) exon of *Dctd*. The sequence in bold is the WT sequence, the sequence in normal font is the observed sequence. A) 5'-region of the deletion. B) 3'-region of the deletion.

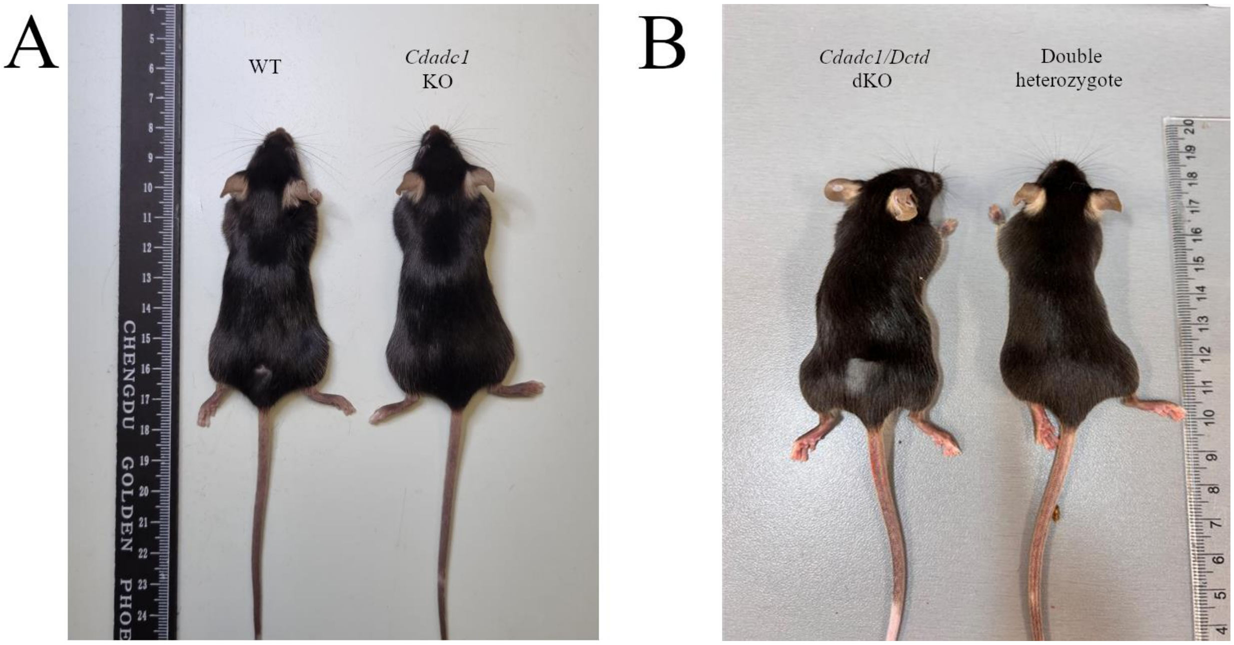

Fig. S27. Lack of overt phenotypes: A) Comparison of WT and *Cdadc1* KO mice. B) Comparison of a *Cdadc1*/Dctd dKO and double heterozygote mice.

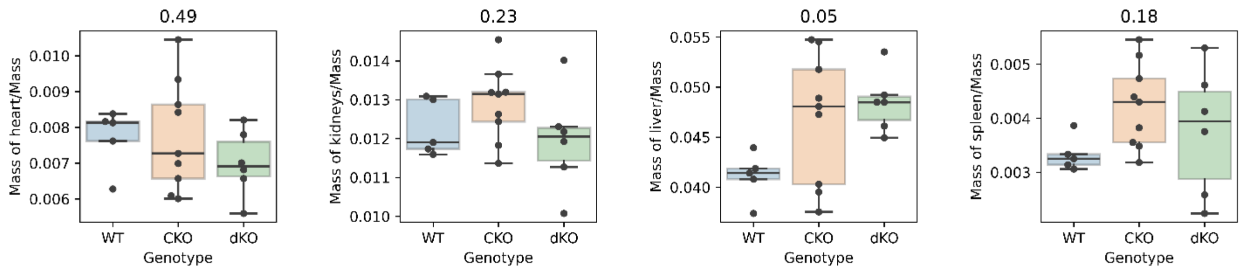

Fig. S28. Organ masses in WT, *Cdadc1* KO (CKO) and *Cdadc1/Dctd* double KO (dKO) mice divided by total body mass to account for the larger body mass in males compared to females.

SI References

1. E. P. Quinlivan, J. F. Gregory, DNA digestion to deoxyribonucleoside: a simplified one-step procedure. *Anal Biochem* **373**, 383–385 (2008).

2. A. Punjani, J. L. Rubinstein, D. J. Fleet, M. A. Brubaker, cryoSPARC: algorithms for rapid unsupervised cryo-EM structure determination. *Nat Methods* **14**, 290–296 (2017).

3. A. Punjani, H. Zhang, D. J. Fleet, Non-uniform refinement: adaptive regularization improves single-particle cryo-EM reconstruction. *Nat Methods* **17**, 1214–1221 (2020).

4. J. Jumper, *et al.*, Highly accurate protein structure prediction with AlphaFold. *Nature* **596**, 583–589 (2021).

5. E. C. Meng, *et al.*, UCSF ChimeraX: Tools for Structure Building and Analysis. *Protein Sci* e4792 (2023). https://doi.org/10.1002/pro.4792.

6. P. Emsley, B. Lohkamp, W. G. Scott, K. Cowtan, Features and development of Coot. *Acta Crystallogr D Biol Crystallogr* **66**, 486–501 (2010).

7. D. Liebschner, *et al.*, Macromolecular structure determination using X-rays, neutrons and electrons: recent developments in Phenix. *Acta Cryst D* **75**, 861–877 (2019).

8. Smart O.S., *et al.*, Grade2 version 1.6.0. Cambridge, United Kingdom: Global Phasing Ltd. (2021).

9. G. E. Truett, *et al.*, Preparation of PCR-quality mouse genomic DNA with hot sodium hydroxide and tris (HotSHOT). *Biotechniques* **29**, 52, 54 (2000).

10. J. A. Harris, A Simple Test of the Goodness of Fit of Mendelian Ratios. *The American Naturalist* **46**, 741–745 (1912).

11. G. F. Maley, A. P. Lobo, F. Maley, Properties of an affinity-column-purified human deoxycytidylate deaminase. *Biochim Biophys Acta* **1162**, 161–170 (1993).

12. H.-F. Hou, Y.-H. Liang, L.-F. Li, X.-D. Su, Y.-H. Dong, Crystal Structures of Streptococcus mutans 2′-Deoxycytidylate Deaminase and Its Complex with Substrate Analog and Allosteric Regulator dCTP·Mg2+. *Journal of Molecular Biology* **377**, 220–231 (2008).

13. Y.-H. Li, *et al.*, Structural basis of a multi-functional deaminase in chlorovirus PBCV-1. *Archives of Biochemistry and Biophysics* **727**, 109339 (2022).

14. A. Marx, A. Alian, The First Crystal Structure of a dTTP-bound Deoxycytidylate Deaminase Validates and Details the Allosteric-Inhibitor Binding Site. *J Biol Chem* **290**, 682–690 (2015).

15. R. Almog, F. Maley, G. F. Maley, R. MacColl, P. Van Roey, Three-Dimensional Structure of the R115E Mutant of T4-Bacteriophage 2‘-Deoxycytidylate Deaminase,. *Biochemistry* **43**, 13715–13723 (2004).

16. F. Sievers, *et al.*, Fast, scalable generation of high-quality protein multiple sequence alignments using Clustal Omega. *Mol Syst Biol* **7**, 539 (2011).

17. D. T. Jones, D. Cozzetto, DISOPRED3: precise disordered region predictions with annotated protein-binding activity. *Bioinformatics* **31**, 857–863 (2015).

18. P. S. Andreev, *et al.*, Spiny chondrichthyan from the lower Silurian of South China. *Nature* **609**, 969–974 (2022).
